# Diversified Chemical Structures and Bioactivities of the Chemical Constituents Found in the Brown Algae Family Sargassaceae

**DOI:** 10.3390/md22020059

**Published:** 2024-01-24

**Authors:** Yan Peng, Xianwen Yang, Riming Huang, Bin Ren, Bin Chen, Yonghong Liu, Hongjie Zhang

**Affiliations:** 1 College of Food Science and Engineering, Lingnan Normal University, Zhanjiang 524048, China; py00_2006@126.com (Y.P.); rb2003227@gmail.com (B.R.); 13724755889@139.com (B.C.); 2 Key Laboratory of Marine Biogenetic Resources, Third Institute of Oceanography, Ministry of Natural Resources, 184 Daxue Road, Xiamen 361005, China; yangxianwen@tio.org.cn; 3 Guangdong Provincial Key Laboratory of Food Quality and Safety, College of Food Science, South China Agricultural University, Guangzhou 510642, China; huangriming@scau.edu.cn; 4 CAS Key Laboratory of Tropical Marine Bio-Resources and Ecology/Guangdong Key Laboratory of Marine Materia Medica, South China Sea Institute of Oceanology, Chinese Academy of Sciences, Guangzhou 510301, China; yonghongliu@scsio.ac.cn; 5 School of Chinese Medicine, Hong Kong Baptist University, 7 Baptist University Road, Kowloon Tong, Kowloon, Hong Kong, China

**Keywords:** brown algae, Fucales, Sargassaceae, secondary metabolites, bioactivity

## Abstract

Sargassaceae, the most abundant family in Fucales, was recently formed through the merging of the two former families Sargassaceae and Cystoseiraceae. It is widely distributed in the world’s oceans, notably in tropical coastal regions, with the exception of the coasts of Antarctica and South America. Numerous bioactivities have been discovered through investigations of the chemical diversity of the Sargassaceae family. The secondary metabolites with unique structures found in this family have been classified as terpenoids, phlorotannins, and steroids, among others. These compounds have exhibited potent pharmacological activities. This review describes the new discovered compounds from Sargassaceae species and their associated bioactivities, citing 136 references covering from March 1975 to August 2023.

## 1. Introduction

Seaweeds, a rich renewable resource, are known to produce numerous complex and diverse secondary metabolites with potent bioactivities [1,2,3,4,5,6,7,8,9,10,11,12,13]. Based on their thallus pigmentation, seaweeds are typically classified into three groups: brown algae (Phaeophyta), green algae (Chlorophyta), and red algae (Rhodophyta). Sargassaceae, a polyphyletic family of brown seaweed, is comprised of the two former families Sargassaceae and Cystoseiraceae [14,15]. This family encompasses a variety of genera, including *Acrocarpia*, *Acystis*, *Anthophycus*, *Axillariella*, *Bifurcaria*, *Carpophyllum*, *Carpoglossum*, *Caulocystis*, *Cladophyllum*, *Coccophora*, *Cystoseira*, *Cystophora*, *Cystophyllum*, *Ericaria*, *Gongolaria*, *Halidrys*, *Hormophysa*, *Landsburǵia*, *Myagropsis*, *Myriodesma*, *Nizamuddinia*, *Oerstedtia*, *Platythalia*, *Sargassum*, *Stolonophorra*, *Scaberia*, and *Turbinaria*, as listed in the algae database [16]. Among these, the genera with the most species are *Sargassum* (977 species) and *Cystoseira* (288 species), followed by *Turbinaria* (53 species) and *Cystophora* (39 species) [16]. Notably, the former two are the most representative genera of this family and have received significant attention, which has resulted in a wealth of publications [4,17,18,19].

Since 1973, studies on Sargassaceae species have experienced rapid growth, leading to the discovery of a multitude of novel compounds with potent bioactivities. Valls and Piovetti summarized 134 new diterpenoids isolated from the former Cystoseiraceae family between 1973 and January 1995 [20], and de Sousa et al. [18] and Gouveira et al. [21] compiled the secondary metabolites isolated from various *Cystoseira* species from 1995 to 2016. Chen and Liu [22] and Rushdi et al. [23] reviewed the chemical constituents of *Sargassum* species and their biological activities from 1974 to 2020. Rushdi et al. [24] also provided an overview of secondary metabolites isolated from *Turbinaria* species between 1972 and 2019. Muñoz et al. [4] summarized the linear diterpenes from *Bifurcaria bifurcata*, emphasizing biosynthetic pathways, biological activities, chemotaxonomy, and ecology. This review attempts to summarize the literature data on the new compounds from the Sargassaceae family and their biological activities.

## 2. Chemistry and Biological Activities of the Compounds from the Sargassaceae Family

**Figure 1:**
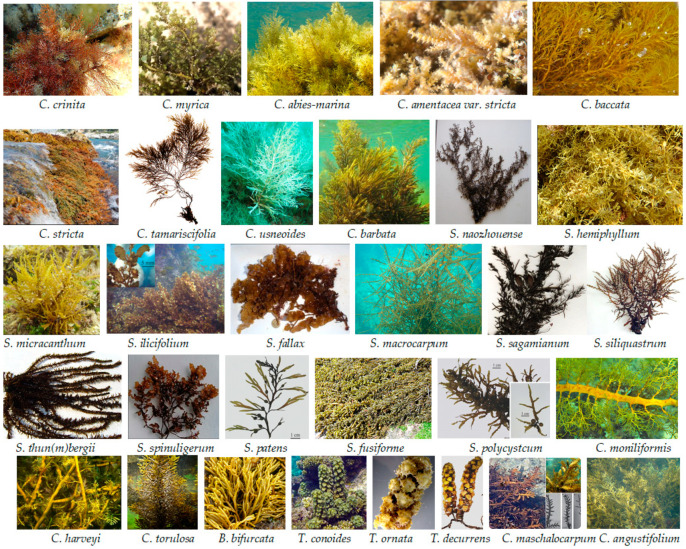
Some Sargassacean species.

Sargassaceae is a family of marine macroalgae comprising over 20 genera and more than 1000 species, and some species are shown in Figure 1. While many genera of this family show a limited distribution, the genera *Bifurcaria*, *Cystophora*, and *Halidrys* display a disjunct distribution [14]. When examining the chemical constituents from Sargassacean species, numerous new structures were obtained, which mainly include terpenoids (encompassing meroterpenoids), phloroglucinol derivatives, steroids, and other types.

### 2.1. Terpenoids

Terpenoids, a class of predominantly secondary metabolites, have been discovered in the Sargassaceae family [25,26]. Specifically, 223 novel terpenoids have been obtained from five different Sargassacean genera, namely *Cystoseira*, *Sargassum*, *Cystophora*, *Bifurcaria*, and *Turbinaria*. Based on the number of isoprene units and the biosynthesis pathway, these isolated compounds can be categorized into monoterpenoids, sesquiterpenoids, diterpenoids, triterpenes, and meroterpenes.

#### 2.1.1. Monoterpenoids

**Figure 2:**
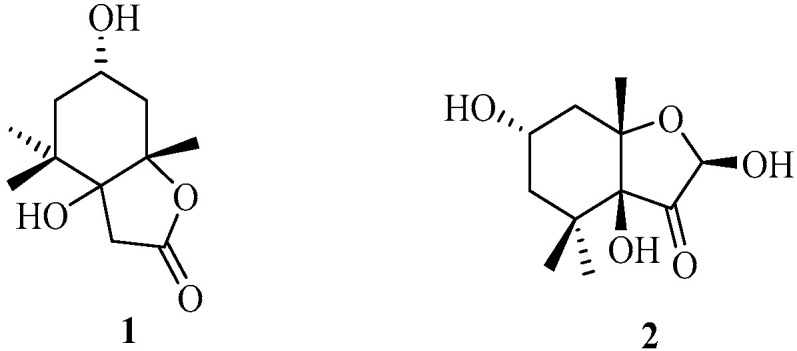
Monoterpenoids isolated from Sargassacean species.

Two new loliolide-type monoterpenoids, schiffnerilolide (**1**) and sargassumone (**2**) (Figure 2), were isolated from the brown algae *C. schiffneri* and *S. naozhouense,* respectively [27,28]. From the biosynthesis aspect, **1** could be derived from isololiolide through oxidation at carbon-carbon double bond [27,29], while **2** may have been formed from loliolide via various reactions, including selective oxidation, specific reduction, and isomerization [28,30].

#### 2.1.2. Sesquiterpenoids

**Figure 3:**
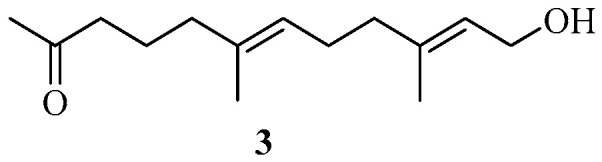
Sesquiterpenoids isolated from Sargassacean species.

A new sesquiterpenoid, oxocrinol (**3**) (Figure 3), was isolated from the Mediterranean alga *C. crinita* [31]. Interestingly, compound **3** was a novel linear terpenoid alcohol, which could potentially originate from farnesol or other possible precursors, such as monoterpenoid and geranylgeraniol [31]. 

#### 2.1.3. Diterpenoids

**Figure 4:**
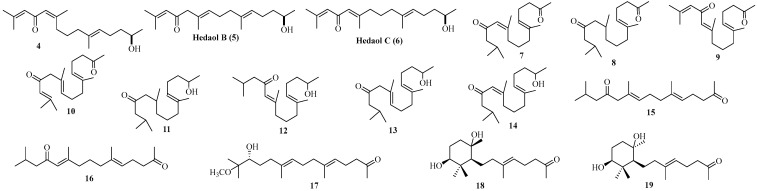
Norditerpenoids isolated from Sargassacean species.

**Figure 5:**
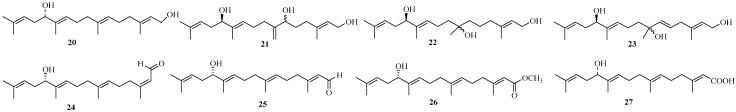
C-12 oxidized linear diterpenoids isolated from Sargassacean species.

**Figure 6:**
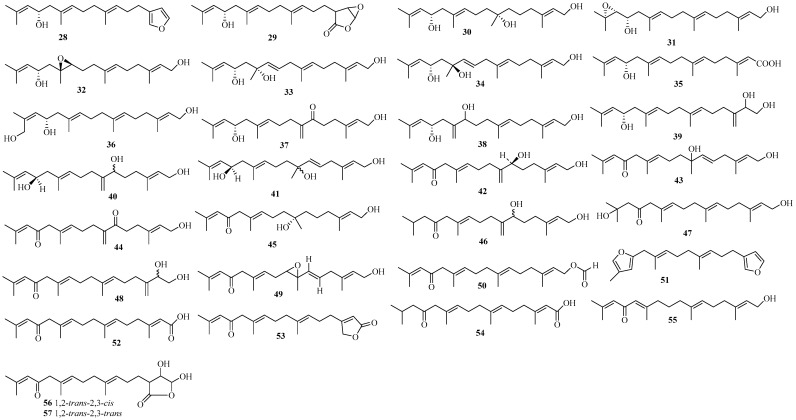
C-13 oxidized linear diterpenoids isolated from Sargassacean species.

**Figure 7:**
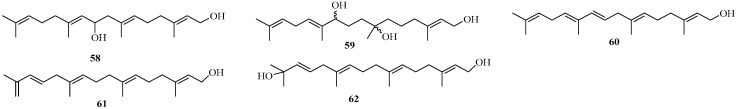
Non C-12/C-13 oxidized linear diterpenoids isolated from Sargassacean species.

**Figure 8:**
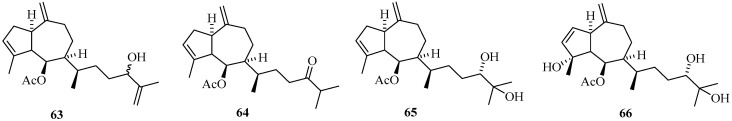
Hydroazulene diterpenes isolated from Sargassacean species.

**Figure 9:**
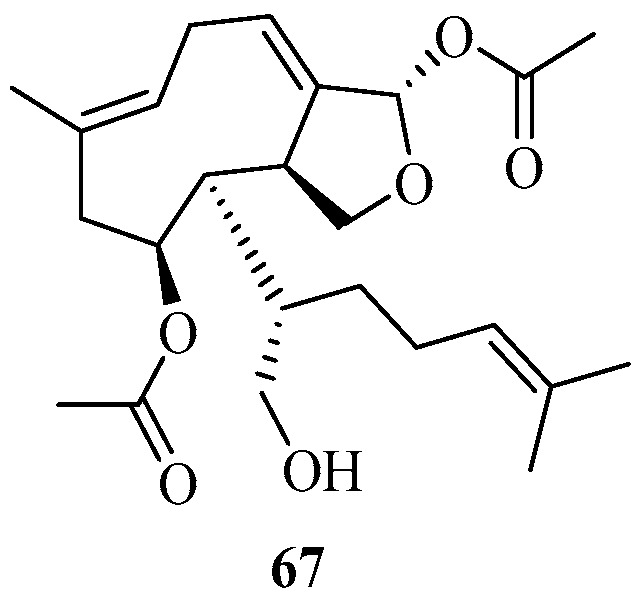
Xenicane diterpenes isolated from Sargassacean species.

Sixty-four new diterpenoids, **4**–**67** (Figure 4, Figure 5, Figure 6, Figure 7, Figure 8 and Figure 9), were isolated from various Sargassacean species. According to the carbon skeletons, these newly isolated compounds were classified into norditerpenoids, acyclic diterpenes, hydroazulene diterpenes, and xenicane diterpenoids. 

##### Norditerpenoids

Sixteen new norditerpenoid compounds (Figure 4), including three bisnorditerpenes and 13 farnesylacetone derivatives, were obtained from the Sargassaceae family. Among them, 13 were from *Sargassum* sp., while one was from *Cystophora* sp.

Compounds **4**–**6**, three novel bisnorditerpene isomers featuring an unusual *α*, *β*-unsaturated ketone skeleton, were isolated from *S. hemiphyllum*, collected from the Heda coast of the Izu Peninsula, Japan. They appeared to originate from the geranyl geraniol precursor and showed low cytotoxicity against P388 cells [32].

Compounds **7**–**16**, novel farnesylacetone derivatives categorized as norditerpenes [33], were isolated from the brown alga *S. micracanthum*, harvested at Kominato, Chiba, Japan [33,34]. From a biosynthetic aspect, these compounds could be formed from geranylgeranylquinones and chromenols through selective oxidation.

Compounds **17**–**19**, also classified as farnesylacetone derivatives belonging to norditerpenoid analogs, were obtained from the brown alga *C. moniliformis*, which was harvested from Port Philip Bay, Australia [35]. Particularly, compounds **18** and **19** were two epimers that were indirectly formed from geranyl acetone [35]. 

##### Acyclic Diterpenoids

Though acyclic diterpenoids are seldom found in nature, they are abundantly found in the brown alga *B. bifurcata* [4]. Notably, 43 new linear diterpenoids (**20**–**62**) (Figure 5, Figure 6 and Figure 7) were obtained from the brown algae *B. bifurcata* and *C. crinita*. Based on their biosynthetic origins, these isolates were categorized into three groups: C-12 oxidized congeners, C-13 oxidized congeners, and non-C-12/C-13 oxidized analogs.

- C-12 Oxidized Congeners

Eight new linear diterpenoids, **20**–**27** (Figure 5), featuring a hydroxyl group at C-12, were isolated from *B. bifurcata* collected from the Atlantic coasts of Morocco between 1984 and 2002 [36,37,38,39,40]. These compounds exhibited close chemical relationships. Interestingly, compound **20** could undergo epoxidation at the C-6/C-7 double bond, followed by dehydration to produce allylic alcohols **21** and **23**, which could be further converted to **22** via a selective reduction at the C-5/C-6 double bond [4,37,38]. In particular, compound **24** was unstable and could slowly transform into its stable isomer **25** at room temperature [39]. Furthermore, **25** could convert into **27**, which could undergo methylation to produce **26** [4,39,40]. Compounds **21** and **22** were tested in vitro for cytotoxicity against the NSCLC-N6 cell line and proved to be active [38].

- C-13 Oxidized Congeners

Fourteen new linear diterpenoids, **28**–**41** (Figure 6), featuring a hydroxyl group at C-13, were isolated from the brown alga *B. bifurcata*, sourced from various geographical origins [41,42,43,44,45]. These compounds could be formed from 13-hydroxygeranylgeraniol, namely eleganediol [4]. Notably, compound **28**, which possesses a furan-3-yl ring formed from eleganediol via terminal cyclization and oxidation, was isolated from the French brown seaweed *B. bifurcata*, along with compound **29** [41]. Compounds **30**–**39** were isolated from the brown seaweed *B. bifurcata,* collected from an intertidal rock pool in County Clare, Ireland [42,43,44]. Compounds **40** and **41**, possibly produced from eleganediol by epoxidation of the C-6/C-7 double bond followed by isomerization to form allylic alcohols, were also obtained from the French brown alga *B. bifurcata* [45]. Compounds **28**, **30**, **31**, and **35** showed cytotoxic, antiprotozoal, and anticancer activity, respectively [41,42,43,44]. 

Sixteen new acyclic diterpenes, **42**–**57** (Figure 6), featuring a ketone function at C-13, were isolated from the brown algae *C. crinita* [46] and *B. bifurcata* [44,45,47,48,49,50]. They could originate from eleganolone. Interestingly, some of these isolates appear to have a close chemical relationship. Specifically, compound **44** could undergo selective reduction of its C-6 ketone group, followed by formation of the corresponding allylic alcohol **42**, which could then convert into **46** [46]. Compounds **46** and **47** are two isomers obtained from the France brown alga *B. bifurcata*, together with compound **48** [45]. Compound **52** could transform into **53** via hydroxylation of C-20 and lactonization, or into **54** following reduction of its C-14/C15 double bond [49]. Compounds **56** and **57** are two eleganolone-type stereoisomers featuring a novel dihydroxy-*γ*-butyrolactone system [50].

- Non C-12/C-13 Oxidized Analogs

Five new linear diterpenoids, **58**–**62** (Figure 7), were isolated the brown alga *C. crinita* [31] and *B. bifurcata* [38,39,40,51]. They are non-C-12/C-13 oxidized congeners, directly or indirectly derived from geranylgeraniol. Among them, compound **58** was isolated from the brown alga *C. crinita*, harvested near Catania, Sicily, Italy [31]. Compound **59**, characterized by a secondary alcohol group at C-10, was isolated from the brown alga *B. bifurcata*, harvested near Oualidia, Morocco [38]. Compound **60**, possessing two conjugated double bonds at C-9 and C-11, was also obtained from the brown alga *B. bifurcata*, collected near Oualidia [39]. Compounds **61** and **62** were isolated from the brown alga *B. bifurcata*, harvested off the Atlantic coast of Morocco [40,51]. Notably, **62** demonstrated potent cytotoxicity to fertilized sea urchin eggs [51].

##### Hydroazulene Diterpenoids 

Four new diterpenoids, **63**–**66** (Figure 8), featuring a hydroazulene skeleton, were isolated from the brown alga *C. myrica*, collected at El-Zafrana, Gulf of Suez, Egypt. Their structures were determined by spectroscopic and chemical techniques. The cytotoxicities of these four compounds were tested in vitro against three different mouse cell lines (NIH3T3, SSVNIH3T3, and KA3IT). The results showed moderate cytotoxicity of all isolates against the cancer cell line KA3IT [52]. 

##### Xenicane Diterpenoids

A new xenicane-type diterpenoid, **67** (Figure 9), was isolated from the organic extract of the intertidal brown alga *S. ilicifolium*, which was harvested from the Gulf of Manner coast, India. This new metabolite, deduced as sargilicixenicane, showed potential anti-inflammatory and antioxidant activities [53].

#### 2.1.4. Nor-Dammarane Triterpenoids

**Figure 10:**
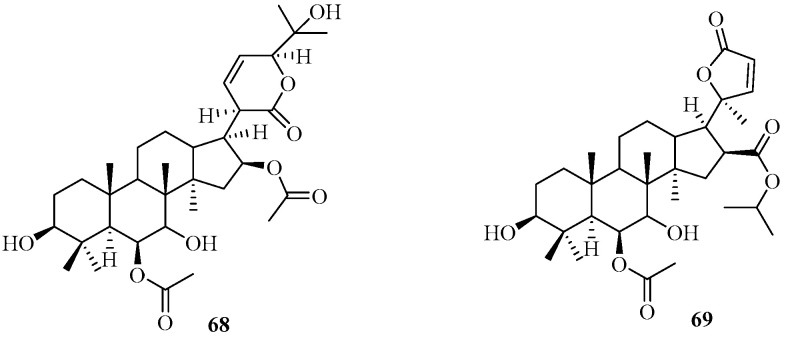
Nor-dammarane triterpenoids isolated from Sargassacean species.

Two new nor-dammarane triterpenes, decurrencylics A-B (**68** and **69**) (Figure 10), were isolated from the brown alga *T. decurrens*, which was harvested from the Mandapam region in the Gulf of Mannar, Peninsular India, India. Their structures were determined by extensive spectra analysis. The two compounds showed potent anti-inflammatory activities [54].

#### 2.1.5. Meroterpenoids

**Figure 11:**
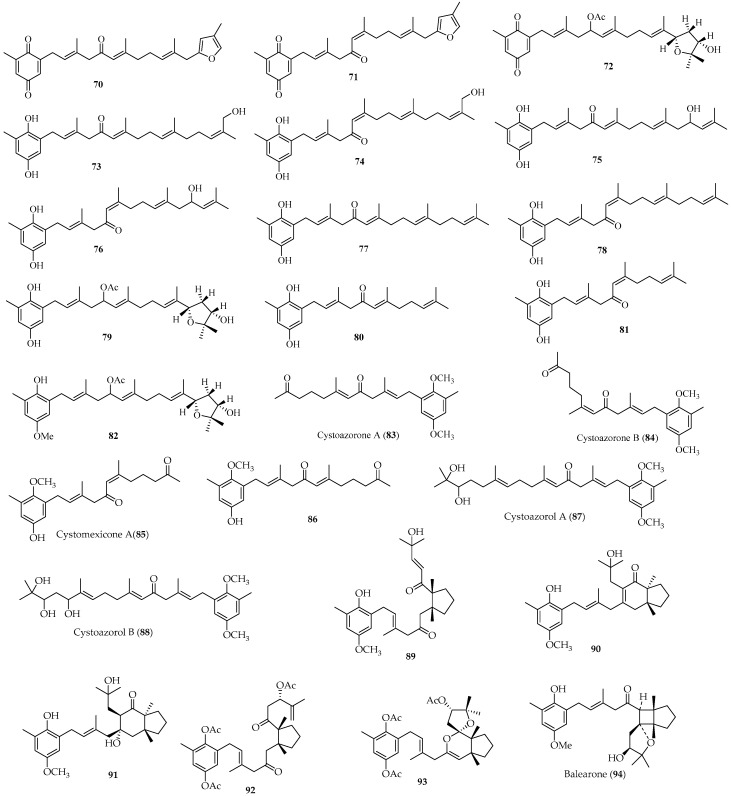

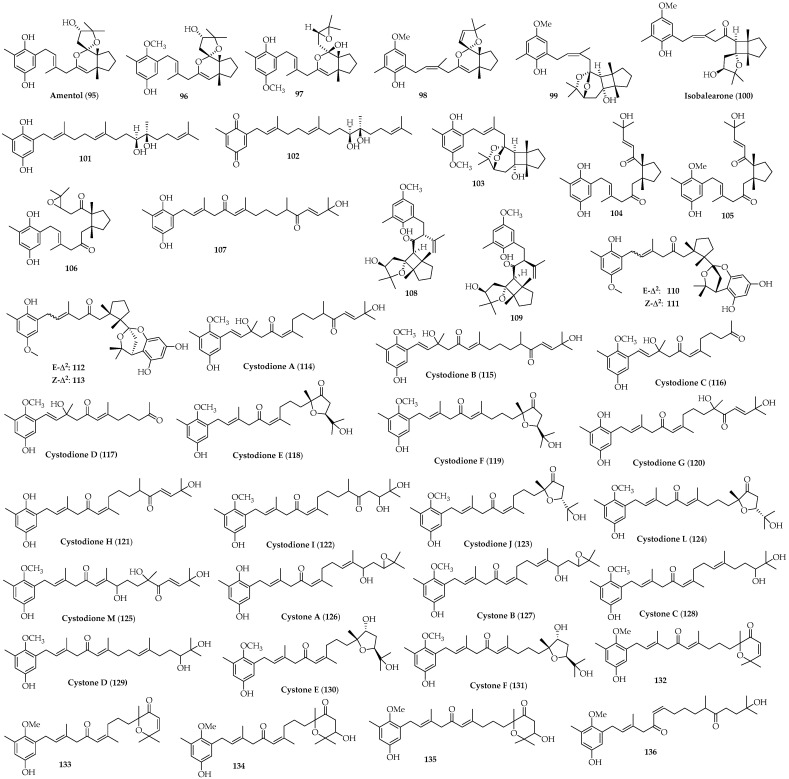

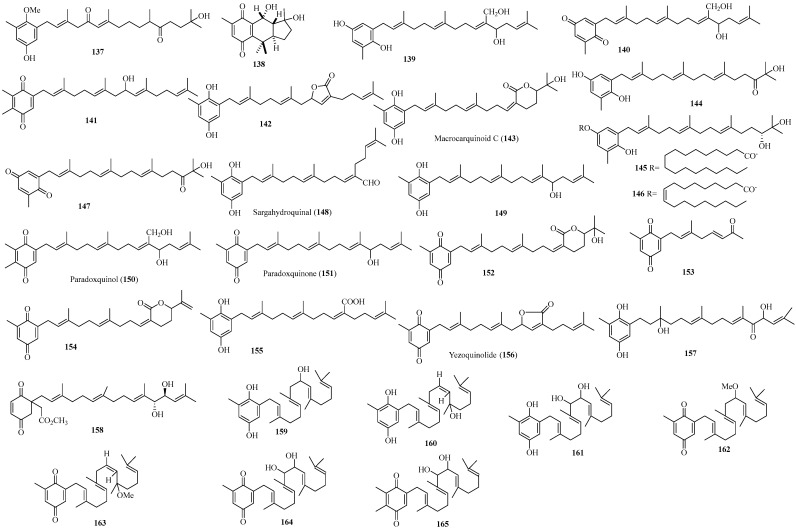
Terpenyl-quinones/hydroquinones isolated from Sargassacean species.

**Figure 12:**
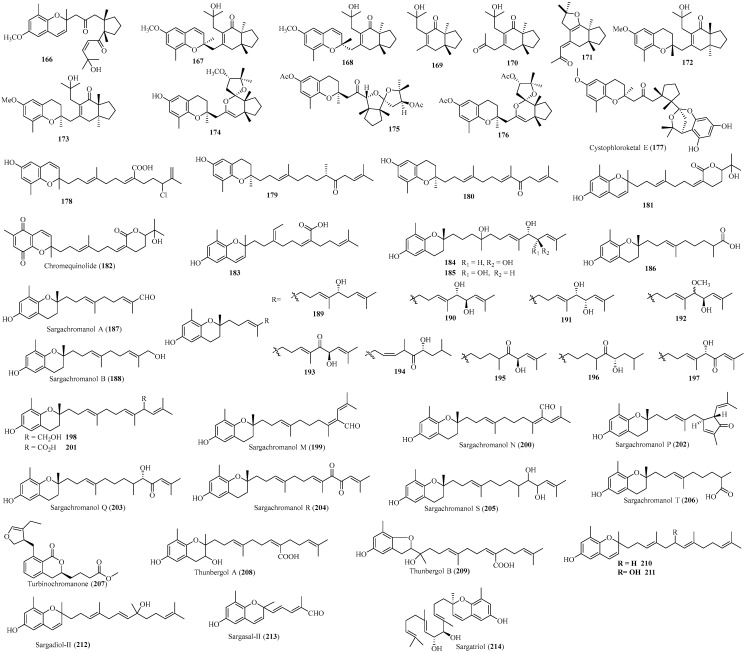
Chromene meroterpenoids isolated from Sargassacean species.

**Figure 13:**
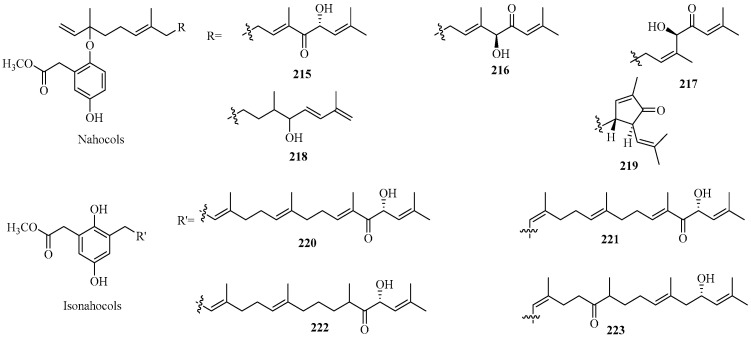
Nahocol/isonahocol meroterpenoids isolated from Sargassacean species.

Meroterpenoids represent another major group of terpene metabolites originating from the Sargassaceae family [6,7,18,55,56,57,58,59,60,61,62,63,64,65,66,67,68,69,70,71,72,73,74,75,76,77,78,79,80,81,82,83,84,85,86]. Notably, 154 new meroterpenoids (**70**–**223**) (Figure 11, Figure 12 and Figure 13), consisting of an aromatic or substituted aromatic nucleus connected to a terpenoid chain with different degrees of oxidation, were isolated from Sargassaceae species [57,58,59,60,61,62,63,64,65,66,67,68,69,70,71,72,73,74,75,76,77,78,79,80,81,82,83,84,85,86]. According to the structural characteristics, meroterpenoids can be classified into terpenyl-quinones/hydroquinone analogs, chromenes, and nahocols/isonahocols. 

##### Terpenyl-Quinones/Hydroquinone Analogs

Ninety-six novel terpenyl-quinones/hydroquinones (**70**–**165**) (Figure 11), which consist of a quinone or hydroquinone nucleus connected to a terpenyl moiety, were isolated from three Sargassacean genera, namely *Cystoseira*, *Sargassum*, and *Cystophora*.

Three novel tetraprenyl-toluquinone derivatives (**70**–**72**), seven new tetraprenyltoluquinols congeners (**73**–**79**), two new triprenyltoluquinol derivatives (**80** and **81**), and one new *O*-methyltoluquinol diterpenoid (**82**) were isolated from two distinct samples of *C. crinita*, one collected from the south coast of Sardinia [57] and another from the French Riviera coasts [58]. Compounds **70**/**71**, **73/74**, **75/76**, **77/78**, and **80/81** belong to five pairs of ∆^6^ stereoisomers and showed antioxidant activities [57]. Particularly, **77** could be formed from **75** via dihydroxylation at C-13′ [57]. Compound **82** could be further converted into **72** and **79** [58]. 

Four new meronorsesquiterpenoids (**83**–**86**) and two new meroditerpenoids (**87** and **88**) were isolated from the brown alga *C. abies-marina* [59,60]. Of them, **83/84** and **85/86** represent two pairs of ∆^6^ diastereomers characterized by a C14 terpenoid side chain, which were possibly formed from the diterpenoid side chain through oxidative degradation [61]. Compounds **87** and **88** contain two methoxyl groups in the aromatic nucleus, which were formed from geranylgeranyltoluquinol via various reaction cascades, such as methylation and/or oxidation [59]. Compounds **83, 84, 87**, and **88** were evaluated for their cytotoxic and antioxidant activities in vitro. The results revealed that **83, 84**, and **87** showed inhibitory activities against Hela cells, while **88** exhibited moderate antioxidant activity against DPPH radicals [59].

A new meroditerpene, 4′-methoxy-2(E)-bifurcarenone (**89**), was isolated from the brown alga *C. amentacea* var. *stricta*, harvested at Le Brusc, France. This new isolate showed cytotoxic effects against the development of the fertilized eggs of sea urchin *Paracentrotus lividus* [62].

Two novel meroditerpenoids (**90** and **91**) were obtained from the brown alga *C. baccata* collected on the Moroccan Atlantic coast. They share the same *trans*-fusion bicyclic [4.3.0] nonane ring system, making the first instance of such a system reported from marine Sargassaceae algae [63]. 

Two new meroditerpenoids, preamentol triacetate (**92**) and 14-epi-amentol triacetate (**93**), were isolated from the acetone extract of an unidentified *Cystoseira* specimen harvested at the Spanish Canary Islands [64]. The two compounds could be formed from geranylgeranyltoluquinol via oxidation and cyclization [65]. 

A novel tetraprenylhydroquinol, balearone (**94**), was isolated from the chloroform extract of the brown alga *C. balearica,* collected at Portopalo, Sicily, Italy. Its chemical structure was deduced by single-crystal X-ray diffraction analysis [66].

Fifteen new tetraprenyl-toluquinol derivatives (**95**–**109**) were isolated from the Mediterranean seaweed *C. stricta*, harvested from three different locations on the Sicilian coasts [67,68,69,70,71,72]. They exhibit structural similarities. Especially, selective methylation of phenolic hydroxyl in **95** could produce the methyl ether **96** [67]. Compounds **99** and **100** are the Z-2-isomers of **103** and **94**, respectively [68,70]. The oxidation of **101** with silver oxide could lead to *p*-benzoquinone **102**, which could also undergo reduction to produce **101** [69]. Compound **104**, derived from **107** via the removal of its acidic proton at C-11 and subsequent formation of the C-11 to C-7 bond, could be converted into **105** by selective methylation, or into **106** via isomerization [71]. Compounds **108** and **109** present two new irregular tetraprenyltoluquinol epimers [72].

Four unique phloroglucinol-meroterpenoid hybrids, named cystophloroketals A–D (**110**–**113**), were isolated from the Mediterranean alga *C. tamariscifolia*, harvested in the Mediterranean Sea near Tipaza, Algeria. They represent the first example of meroterpenoids with a 2,7-dioxabicyclo [3.2.1] octane unit fused to a phloroglucinol. Their antifouling activities were assessed against several marine species involved in the biofouling process, and the results showed that they were active [73].

Twenty-two new meroterpenoids, namely cystodiones A–M (**114**–**125**), cystones A–F (**126**–**131**), usneoidones E and Z (**132** and **133**), and usneoidoles Z and E (**134** and **135**), were isolated from the brown alga *C. usneoides* collected from the Moroccan, Spanish, and Portuguese coasts [74,75,76,77]. Of which, **114**, **115**, and **118**–**135** consist of a toluquinol core and a diterpenoid chain with various oxygenated functionalities and unsaturation, while **116** and **117** consist of a C_14_-side chain attached to an *O*-methyltoluquinol ring [74,75,76,77]. Interestingly, compounds **114/115**, **116/117**, **118/119**, **123/124**, **128/129**, **130/131**, **132/133**, and **134/135** form eight pairs of ∆^6^ stereoisomers. Compounds **114**–**117** displayed antioxidant activities in the ABTS radical-scavenging assay, along with **120**–**131** [74,75,76,77]. Compounds **120**, **125**, and **128** also showed significant inhibitory activities on production of the proinflammatory cytokine TNF-α in LPS-stimulated THP-1 human macrophages [75]. Furthermore, compounds **132**–**135** exhibited antitumor and antiviral activities [76,77].

A pair of novel tetraprenyltoluquinol isomers, **136** and **137**, were isolated from the brown alga *C. sauvageuana*, collected at Aci Castello, Sicily, Italy. It was determined that **136** could be converted into **137** after photoisomerization [78]. 

A novel, linearly fused 6,6,5-tricyclic geranyltoluquinone, pycnanthuquinone C (**138)**, was isolated from the acetone extract of the Western Australian marine brown alga *Cystophora harveyi*. This marks the second report of prenylated quinone with a linear 6,6,5-cyclic skeleton from marine organisms [79].

Two new meroditerpenoids, fallahydroquinone (**139**) and fallaquinone (**140**), were isolated from the brown alga *S. fallax*, collected from Port Philip Bay, Victoria, Australia [80]. Compound **140** is likely to be an artifact compound, as it could be produced from **139** by oxidation upon exposure to air. The absolute stereochemistry for **139** and **140** could not be established, owing to their instability and rapid decomposition. The two isolates displayed weak antitumor activities in a P388 assay [80].

Three new meroterpenoids, macrocarquinoids A–C (**141**–**143**), were isolated from the EtOH extract of the brown alga *S. macrocarpum*, harvested on the coast of Tsukumo Bay, Japan. Compound **142** possesses a *γ*-lactone ring at C-9′ to C-11′ and C-18′ of the terpenyl chain, while **143** has a *δ*-lactone ring at C-11′ to C-14′ and C-18′ [81]. All of these compounds showed inhibitory activity against AGE that were either comparable to, or more potent than, activity of aminoguanidine, which was used as a positive control [81]. 

Four new plastoquinones **144**–**147** were isolated from the brown alga *S. micracanthum*, collected from the Toyama Bay coast of Japan. Their structures were determined by spectroscopic analysis and chemical conversions. Compounds **144**–**146** showed both antioxidant and cytotoxic activities [82]. 

Four new meroditerpenoids—sargahydroquinal (**148**), paradoxhydroquinone (**149**), paradoxquinol (**150**), and paradoxquinone (**151**)—were isolated from the brown alga *S. paradoxum*, collected from Governor Reef near Indented Head, Port Philip Bay, Australia. They consisted of a diterpenoid chain attached to hydroquinone or *p*-benzoquinone rings. Their structures were determined by spectroscopic techniques. Particularly, **148** was identified by HPLC-NMR and HPLC-MS, coupled with comparison with the known compound due to its instability. Compounds **149**–**151** showed weak antibacterial activities against *Streptococcus pyogenes* [83].

Three new sargaquinoic acid derivatives, 15′-hydroxysargaquinolide (**152**), (2′E,5′E)-2-methyl-6-(7′-oxo-3′-methylocta-2′,5′-dienyl)-1,4-benzoquinone (**153**), and 15′-methylenesargaquinolide (**154**), and two new plastoquinone analogs, sargahydroquinoic acid (**155**) and yezoquinolide (**156**), were isolated from the brown algae *S. sagamianum* [84] and *S. sagamianum* var. *yezoense* [85]. Noticeably, **153** and **154** are a selectively oxidized analog and a dehydration derivative of **152**, respectively [83]. Compound **155** is a hydroquinone derivative of sargaquinoic acid [53], while **156** features an *α*, *β*-unsaturated *γ*-lactone moiety, marking the first example of a plastoquinone with a butenolide unit [85]. Compounds **152** and **153** showed antibacterial activities and cytotoxicities against Hela S3 cells [84]. 

Two new meroditerpenoids (**157** and **158**) were isolated from the brown alga *S. siliquastrum*, collected from Jeju Island, Korea [86]. Compound **157**, a derivative of sargahydroquinoic acid, exhibited significant radical-scavenging activity as well as slight inhibitory activity against isocitrate lyase from *Candida albicans*. The stereochemistry at C-13′ of **157** remained uncertain due to the limited quantity. Compound **158**, representing the first reported meroditerpenoid with a modified dihydroquinone unit from marine brown algae, exhibited weak activity against transpeptidase sortase A from *Staphylococcus aureus* [86]. Interestingly, **158** was presumed to be a biosynthetic precursor of nahocols and isonahocols, based on a 1,3-migration of its methyl acetate group. 

Seven new geranylgeranylbenzoquinone derivatives (**159**–**165**) were separated from the Japanese marine alga *S. tortile* harvested at Awa-Kominato, Chiba, Japan. These isolates consist of a hydroquinone or benzoquinone core linked to a diterpenoid moiety. Among them, compounds **159**/**160** and **162/163** constitute two pair of isomers. Compound **161** could be converted into quinone **164** by selective oxidation [87].

##### Chromenes

Forty-nine new chromene meroterpenods (Figure 12) were isolated from certain species of Sargassaceae. Their structures are similar to that of vitamin E.

A new chromene meroditerpene (**166**) was isolated from the brown alga *C. amentacea* var. *stricta* mentioned above. It is a derivative of 4′-methoxy-2(*E*)-bifurcarenone originated from the same species [62]. 

Two novel chromene meroditerpenoid isomers (**167** and **168**) and their derivatives (**169**–**171**), together with two new chromane meroditerpenoid epimers (**172** and **173**), were isolated from the brown alga *C. baccata* and *S. muticum* [63,87,88,89]. Among them, compounds **167**–**171** share the same *trans*-fused carbon skeleton, marking the first report of such a structure in the Sargassaceae family [63]. Compounds **172** and **173** also possess the same *trans*-fused bicyclic system and were found to exhibit photodamage attenuation effects [89,90]. Compounds **168**, **169**, and **171** showed antifouling activities against the settlement of certain macroalgae, the growth of microalgae, and the activities of mussels [63]. 

Three new chromane meroditerpenes (**174**–**176**) were isolated from the previously mentioned unidentified *Cystoseira* specimen. Due to their inherent instability, **175** and **176** were only obtained in the acetate form. In particular, **175** represented the first example of meroditerpene containing a newly rearranged structure, featuring a novel ether linkage in the diterpene chain. The structure is likely formed from **176** via an oxidation process of the enol-ether system, followed by rearrangement [64].

A new phloroglucinol-meroditerpenoid hybrid (**177**), consisting of a chromane meroditerpenoid linked to a phloroglucinol through a 2,7-dioxabicylo [3.2.1] octane unit, was isolated from the brown alga *C. tamariscifolia* mentioned above. This isolate showed moderate to weak antifouling activities against several marine colonizing species such as bacteria, fungi, micro- and macroalgae [73].

A new chromene meroditerpenoid, fallachromenoic acid (**178**), featuring a carboxylic group and a chlorine atom, was isolated from the brown alga *S. fallax* described above. Its absolute configuration could not be assigned due to its instability [80]. Compound **178** showed weak antitumor activity against P388 murine leukemia cells [80].

Two new chromane meroterpenoids (**179** and **180**) were obtained from the brown alga *S. micracanthum*, harvested on the Toyama Bay coast, Japan. Their structures were determined by extensive spectroscopic analysis and chemical conversion [91].

Two new chromene meroditerpenoids (**181** and **182**), characterized by a lactone ring, were isolated from the Japanese alga *S. sagamianum* mentioned above [84]. Their structures were determined by extensive spectrometric analysis and comparison with published data. Particularly, **181** exhibited antibacterial and weak cytotoxic activities [84].

Twenty-four chromene meroterpenoids (**183**–**206**) were isolated from two distinct samples of *S. siliquastrum*, one collected from the seashore of Pusan [92], and another from Jeju Island (Korea) [93,94,95,96]. Among them, **186**–**188** and **206** contain a linear triprenyl moiety, while the rest possess a tetraprenyl moiety [93,94]. Notably, **198**–**201** contained a rearranged tetraprenyl carbon skeleton, while **202** had a cyclized tetraprenyl chain, reported for the first time [94]. Compounds **183**–**202**, **205**, and **206** showed antioxidant activities [92,93,94,96], while **193** and **201** were found to display inhibitory activities toward butylcholine esterase [94]. Additionally, **203** and **204** exhibited cytotoxic activities against AGS, HT-29, and HT-1080 cell lines [95].

A novel furanyl-substituted isochromanyl derivative, turbinochromanone (**207**), was isolated from the ethyl acetate-methanolic extract of the brown seaweed *Turbinaria conoides*, collected from the coasts of Peninsular India. Compound **207** exhibited potential attenuation properties against 5-lipoxygenase and cyclooxygenase-2-enzyme. Furthermore, its antioxidant properties supported its potential use as an anti-inflammatory agent [97]. 

Two new tetraprenyltoluquinol isomers, thunbergol A (**208**) and B (**209**), were obtained from the brown alga *S. thunbergii* collected along the Busan coast of Korea. The two compounds showed antioxidant effects against DPPH radical and authentic/induced ONOO^−^ [98].

Four new chromene compounds (**210**–**213**), along with a new isoprenoid chromenol (**214**), were isolated from two distinct samples of *S. tortile*, one collected from the coast of Tanabe Bay, Japan [99], and the other from Wakasa Bay, Fukui Prefecture, Japan [100,101]. Compounds **210**–**213** showed cytotoxic activities toward cultured P-388 lymphocytic leukemia cells [99].

##### Nahocols/Isonahocols

Five new nahocols (**215**–**219**) and four novel isonahocols (**220**–**223**) were isolated from the brown alga *S. siliquastrum* mentioned above [86,102]. Their structures are shown in Figure 13. They share structural similarities to **158** [86]. Especially, **219** contains a cyclopentenone moiety, the characteristic cyclization pattern of which has only been reported for the second time in marine algae. All of them exhibited radical-scavenging activity against DPPH free radicals. Furthermore, isonahocols **220**–**223** showed a 100-fold increase in radical-scavenging activities compared with nahocols **215**–**219**, indicating the crucial role of the phenolic group in DPPH radical scavenging activity. In addition, **215**–**219** showed still-weak activities against isocitrate lyase from *Candida albicans*, while **220**–**223** exhibited inhibitory effects on transpeptidase sortase A derived from *Staphylococcus aureus*. 

### 2.2. Phloroglucinols

To date, numerous phloroglucinol derivatives have been identified in brown seaweed species [103,104]. Notably, some new phloroglucinols were obtained from Sargassaceae species [105,106,107,108,109,110,111,112,113,114,115,116]. Based on the number of phloroglucinol units, phloroglucinols may be conveniently classified into monomeric phloroglucinols and phlorotannins. 

#### 2.2.1. Monomeric Phloroglucinols

**Figure 14:**
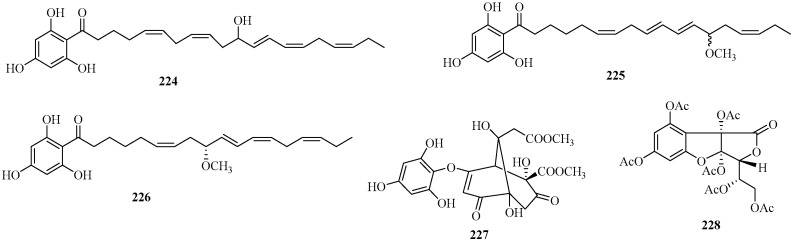
Monomeric phloroglucinols isolated from Sargassacean species.

Five new monomeric phloroglucinols, **224**–**228** (Figure 14), were isolated from the brown algae *S. nigrifoloides*, *S. micracanthum*, and *S. spinuligerum* [105,106,107]. Among them, compounds **224**–**226** are classified as acyphloroglycinols, and they were isolated from the brown alga *S. nigrifoloides* collected at Nanji Island of Zhejiang, China [105]. These three compounds exhibited inhibitory activities against CDK5 and GSK3β [105].

Compound **227**, consisting of a hydroxyphloroglucinol unit and a sargassumketone moiety, was obtained from the brown alga *S. micracanthum*, collected at Wando County, Korea. It showed radical-scavenging activity against ABTS^+^ radicals [106].

Compound **228**, containing a phloroglucinol unit and an ascorbic acid moiety, was isolated from the ethanolic extract of the brown alga *S. spinuligerum* as a novel phloroglucinol derivate. Its stereochemistry was determined through NOE experiments and molecular modeling [107].

#### 2.2.2. Phlorotannins

Phlorotannins, a major class in the unique phloroglucinol-based polyphenols, were predominantly found in the Sargassaceae family [103,104]. These compounds were mainly isolated as their acetates due to their instability. Over recent decades, a great number of phlorotannins have been isolated from various Sargassacean species [108,109,110,111,112,113,114,115,116]. According to the types of linkages between the phloroglucinol units, phlorotannins have been systematically categorized into groups such as fucophlorethols, hydroxyphlorethols, carmalols, phlorethofuhalols, and fuhalols, among others. 

##### Fucophlorethols

**Figure 15:**
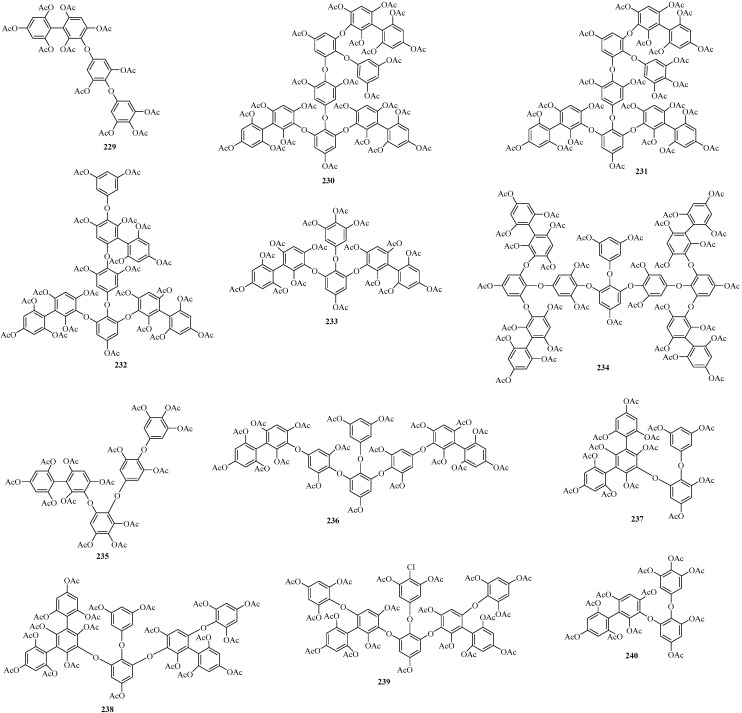

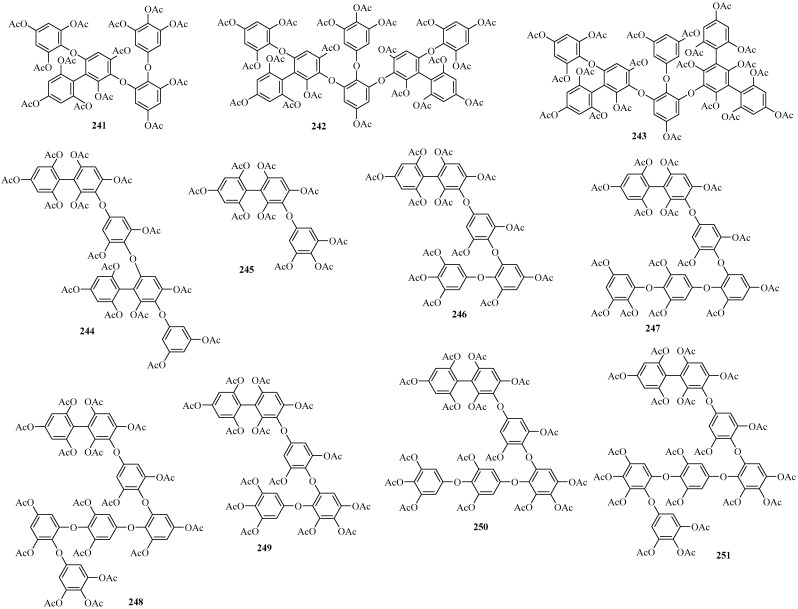
Phloroglucinol derivatives belonging to the class of fucophlorethols.

Twenty-three new phloroglucinol derivatives (**229**–**251**) (Figure 15), belonging to the class of fucophlorethols with three to fourteen rings, were isolated from three distinct Sargassaceae species, namely *Carpophyllum maschalocarpum*, *S. spinuligerum*, and *Cystophora torulosa*. Among these, **229**–**234** were obtained from the brown alga *C. maschalocarpum* collected at Torbay, north of Auckland, New Zealand [108]. Interestingly, **234** is the largest fucophlorethol, characterized by 14 phloroglucinol units. Due to the presence of extra hydroxyl groups, **229**, **231**, and **233** were also categorized as hydroxyfucophlorethols.

Compounds **235**–**239** were isolated from the brown alga *S. spinuligerum*, collected from Wangaparoa Island, district Auckland, New Zealand [109]. Notably, **238** and **239** were once again obtained from the brown alga *C. torulosa*, collected at Whangaparoa, New Zealand [109]. Interestingly, **239** was found as a chlorine-containing fucophlorethol.

Compounds **240**–**251** were obtained from the brown algae *C. torulosa* and *S. spinuligerum* harvested at Whangaparoa, New Zealand [109,110]. Among them, **240**–**242** and **245**–**251** contain additional hydroxy groups, leading to their classification as hydroxyfucophlorethols as well [110,111]. Compounds **243** and **244**, however, are bis-fucophlorethols that lack a 1,2,3-triphenoxy-5-acetoxybenzene unit [110].

##### Hydroxyphlorethols

**Figure 16:**
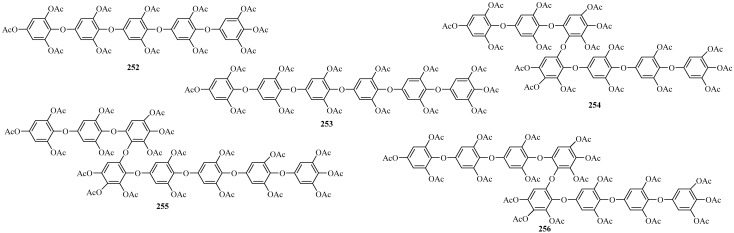
Phloroglucinol derivatives belonging to the class of hydroxyphlorethols.

Five new phloroglucinol derivatives belonging to the class of hydroxyphlorethols, **252**–**256** (Figure 16), were isolated from two *Carpophyllum* species, namely *C. maschalocarpum* and *C. angustifolium* [112,113]. Specifically, **252** and **253**, which contain an additional hydroxyl group, were isolated from the brown alga *C. maschalocarpum* collected at Torbay, north of Auckland [112]. 

Compounds **254**–**256** feature three additional hydroxyl groups as well as two 1,2-diphoxylated 3,4,5-triacetoxybenzene rings linked by an ether bond, leading to their designation as trihydroxyphlorethols. All of them were isolated from the brown alga *C. angustifolium* harvested at Panetiki Island, Cape Rodney [113].

##### Carmalols

**Figure 17:**
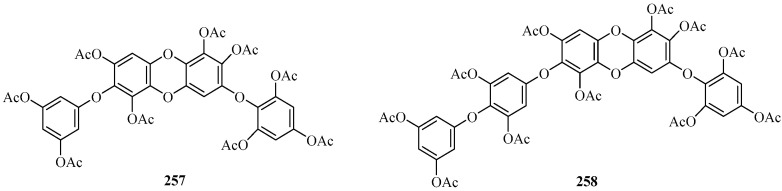
Phloroglucinol derivatives belonging to the class of carmalols.

Two new phloroglucinol derivatives belonging to the class of carmalols (**257** and **258)** (Figure 17) were isolated from the brown alga *C. maschalocarpum* mentioned above [112,114]. Compound **257** contains two phloroglucinol units and an additional hydroxyl group, and it was named diphlorethohydroxycarmalol nonaacetate. Meanwhile, **258**, which possesses three phloroglucinol units and one additional hydroxyl group, was designated as triphlorethohydroxycarmalol undecaacetate [114].

##### Phlorethofuhalols

**Figure 18:**
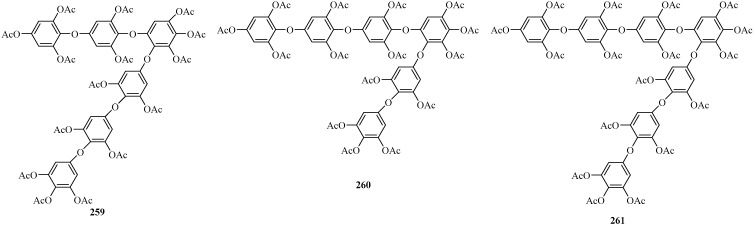
Phloroglucinol derivatives belonging to the class of phlorethofuhalols.

Three new phloroglucinol derivatives (**259**–**261**) (Figure 18), which are part of the phlorethofuhalol class containing an increased number of 1,4-diphenoxylated 3,5-diacetoxy-benzene rings compared with their corresponding fuhalol counterparts, were isolated from the brown alga *C. maschalocarpum*. Among them, **259** and **260** were two isomers composed of six phloroglucinol units linked by ether bonds, whereas **261** consisted of seven phloroglucinol elements linked by ether bonds and contained one additional 1,4-diphenoxylated 3,5-diacetoxybenzene moiety [114].

##### Fuhalols and Others

**Figure 19:**
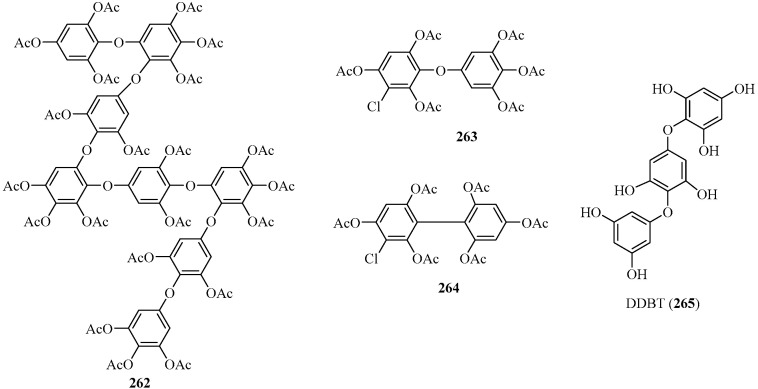
Phloroglucinol derivatives belonging to the class of fuhalols and others.

A new phloroglucinol derivative belonging to the class of fuhalols, **262** (Figure 19), together with two new phlorotannins with a chlorine atom (**263** and **264**), were isolated from the brown alga *C. angustifolium*, collected at Panetike Island/Cape Rodney/New Zealand [115]. Among them, **262** consists of eight phloroglucinol units linked by ether bonds and contains additional hydroxyl groups. Compound **263** is a chlorinated bifuhalol derivative, whereas **264** is a chlorinated difucol derivative. 

In addition, a new phloroglucinol derivative, DDBT (**265**) (Figure 19), was isolated from the brown alga *S. patens*, harvested from the coast of the Noto Peninsula, Japan. This compound showed inhibitory effects against α-amylase and α-glucosidase [116]. 

### 2.3. Steroids

**Figure 20:**
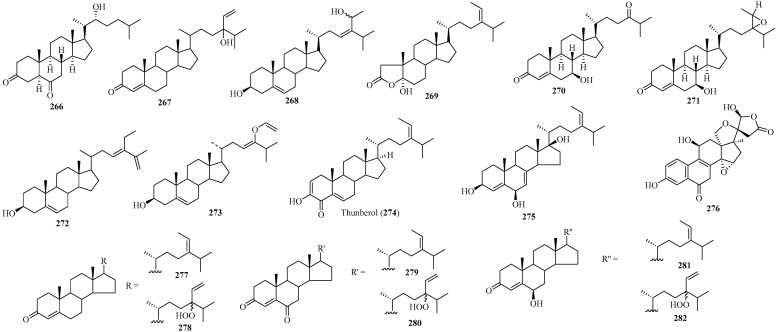
Steroids isolated from the family Sargassaceae.

Steroids are another class of unique metabolites discovered in the Sargassaceae family. Seventeen new sterols (**266**–**282**) (Figure 20) were isolated from various species of Sargassaceae [117,118,119,120,121,122,123,124,125]. Interestingly, they are C_23_-, C_27_-, and C_29_- steroids, characterized by keto and hydroxy groups. Among these steroids, one was obtained from *Cystoseira* sp., eight from *Sargassum* sp., and eight from *Turbinaria* sp. 

Compound **266**, a C_27_-brassinosteroid with two keto groups and a hydroxy group, was isolated from the brown alga *C. myrica*, harvested from the region of Fayed, Egypt. It represented the first report of brassinosteroid analogs derived from seaweed. Compound **266** showed cytotoxic effects against HEPG-2 and HCT116 cell lines [117].

Compound **267**, a C_29_-steroid with an *α*, *β*-unsaturated carbonyl group and a tertiary hydroxyl group, was isolated from the brown alga *S. asperifolium*, collected at Hurghada, Egypt. From a biosynthetic perspective, **267** could potentially be derived from saringosterol via an oxidation process involving 3*β*-OH, followed by the formation of an *α*, *β*-unsaturated ketone [118].

Compounds **268** and **269**, two polyoxygenated steroids, were isolated from the brown alga *S. carpophyllum*, harvested from the coasts of the South China Sea in Beihai, China. Specifically, **268** is a C_29_-polyoxygenated steroid, while **269** is a C_27_-dinorsteroid, representing only the second example of ring A-dinorsteroid analogs found in natural organisms. Both compounds could induce morphological abnormalities of *Pyricularia oryzae* mycelia. In addition, **268** exhibited cytotoxic activity against HL-60 cell lines [119].

Compounds **270** and **271** are two cholestane-type sterols, each featuring an *α*, *β*-unsaturated ketone moiety. Among them, **270** is a C_27_-steroid, while **271** is a C_29_-steroid. Both were isolated from the brown alga *S. fusiforme*, harvested from Anhui Bozhou Xiancheng Pharmaceutical Limited Company of China. Their absolute configurations were determined by comparing the calculated and experimental ECD spectra [120]. 

Compound **272**, a stigmastane-type sterol characterized by three double bonds and one hydroxyl group, was isolated from the brown alga *S. polycystcum*, collected from the North China Sea, China [121].

Compound **273**, a tri-unsaturated C_29_-sterol with a 3*β*-hydroxy-Δ^5^-steroid skeleton and a vinyloxy group, was isolated from the brown alga *S. thumbergii*, harvested at Muroran, Japan. Its structure was determined by combining NMR spectroscopy and chemical conversion [122].

Compound **274**, a C_29_-sterol with a 3-hydroxy-2,5-dien-4-carbonyl fragment, was isolated from the brown alga *S. thunbergii*, harvested along the coasts of Nanji Island in the East China Sea of China. It was the first sterol example discovered to contain a 3-hydroxy-2,5-dien-4-carbonyl moiety. Compound **274** showed significant inhibitory activity against PTP1B with an IC_50_ of 2.24 μg/mL [123].

Compounds **275**–**282**, which are oxygenated steroids, were isolated from two separate samples of *Turbinaria conoides*, one collected at Salin Munthal (India) [124] and another at the coast of Kenting (Taiwan). Notably, **276** is identified as a cardenolide-type C_23_ steroid with an aromatic ring, while the remaining compounds are either stigmasterol or fucosterol derivatives, comprised of 29 carbons. Compounds **275** and **276** showed antimicrobial activities [124], whereas **279**–**282** exhibited cytotoxic effects against cancer cell lines P-388, KB, A-549, and HT-29 [125].

### 2.4. Others

**Figure 21:**
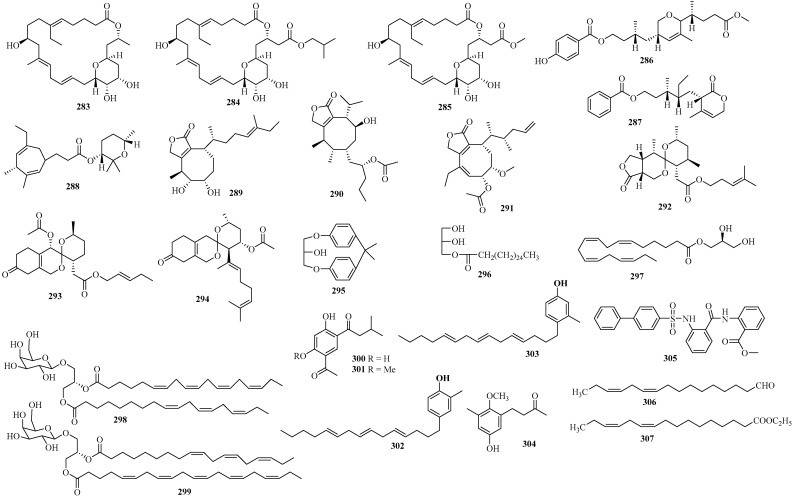
Other types of compounds isolated from Sargassacean species.

Apart from producing an abundance of unique terpenoids, phloroglucinols, and steroids, Sargassaceae species also generate a variety of other metabolites, including macrocyclic lactones, pyran derivatives, furanones, spiroketals, glycerol derivatives, phenol derivatives, amide derivatives, and lipids (Figure 21). 

Three new macrolide compounds, conoidecyclics A–C (**283**–**285**), along with three novel 2H-pyranoids (**286**–**288**), were isolated from the brown alga *T. conoides*, harvested from the Gulf of Mannar, India [126,127]. These isolates showed anti-inflammatory and radical scavenging activities. Specifically, compounds **283**–**285** also exhibited antihypertensive and antidiabetic activities [126].

Three new terpenic cyclooctafuranones, turbinafuranones A–C (**289**–**291**), together with three novel 6,6-spiroketals, spirornatas A–C (**292**–**294**), were isolated from the marine alga *T. orata*, collected from the Gulf of Manner of India [128,129]. The six compounds showed scavenging activities against DPPH and ABTS radicals. Notably, **289**–**291** also exhibited in vitro antidiabetic properties [128], while **292**–**294** showed antihypertensive activities [129]. 

Five new glycerol derivatives, identified as **295**–**299**, were isolated from three different *Sargassum* species [130,131,132]. Among them, **295** and **296** were identified from *S. parvivesiculosum* in Sanya, China, **297** was obtained from *S. sagamianum* on Jeju Island, Korea [131], and **298** and **299** were derived from *S. thunbergii* in the West Sea, Korea [132]. Particularly, **296** and **297** were determined to be monoglycerides, whereas **298** and **299** were glycolipids. Compound **297** exhibited inhibitory activities against COX-2 and sPLA2-IIA [131].

Two novel resorcinols, 1-(5-acetyl-2,4-dihydroxyphenyl)-3-methylbutan-1-one (**300**) and 1-(5-acetyl-2-hydroxy-4-methoxyphenyl)-3-methylbutan-1-one (**301**), were isolated from the brown alga *S. thunbergii*, supplied by the Guanghua Algae Company in Weihai, Shandong, China. Their structures were determined by extensive spectrometric analysis [133].

Two new aryl cresol isomers (**302** and **303**) were isolated from the brown alga *S. cinereum*, harvested along the coasts of the Red Sea in Hurghada, Egypt. Interestingly, the two isolates showed antiproliferative activities against certain cancer cell lines and inhibitory effects against 5-LOX and 15-LOX, the enzymes that have a vital effect on the viability of tumor cells [134].

A novel ketone hybrid of mix biogenesis (**304**), consisting of a four-carbon chain attached to a hydroquinol ring, was isolated from the aforementioned brown alga *C. abies* [60]. Its structure was determined by spectroscopic analysis, including NMR, MS, and UV.

A new amide derivative, sargassulfamide A (**305**), was obtained from the brown alga *S. naozhouense*, harvested from the Leizhou Peninsula, China. Its structure was established by spectrometric analysis and single-crystal X-ray diffraction [135].

Two new unsaturated lipids, (10*Z*,13*Z*)-hexadeca-10,13-dienal (**306**) and *E*thyl-(10*Z*,13*Z*)-hexadeca-10,13-dienoate (**307**), were isolated from the brown alga *C. barbata*, harvested from Salses, France. Compound **306** showed anticancer effects against P388 cells in mice at 40 mg/kg [136]. 

## 3. Conclusions

The merging of the former Cystoseiraceae and Sargassaceae families has resulted in Sargassaceae becoming the largest family in Fucale. To date, more than 60 species of Sargassaceae have been chemically studied, leading to the identification of more than 400 metabolites. Based the available literature, this review summarizes a total of 307 new compounds obtained from 44 Sargassaceae species spanning six genera, and newly discovered compounds derived from the 44 species collected from diverse locations along the Tunisian, Chinese, Italian, Japanese, Australian, Moroccan, Irish Atlantic, Spanish, French, Indian, Egyptian, Portuguese, Algerian, Korean, and New Zealand coasts (Table 1). These include 223 terpenoids, 42 phloroglucinols, 17 steroids, and 25 other types of compounds.

**Figure 22:**
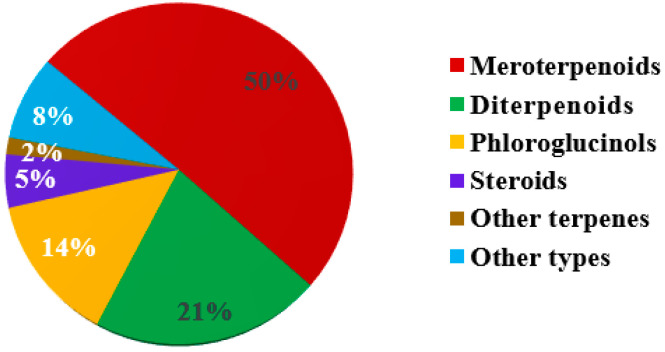
Distribution of compounds from Sargassacean species.

**Figure 23:**
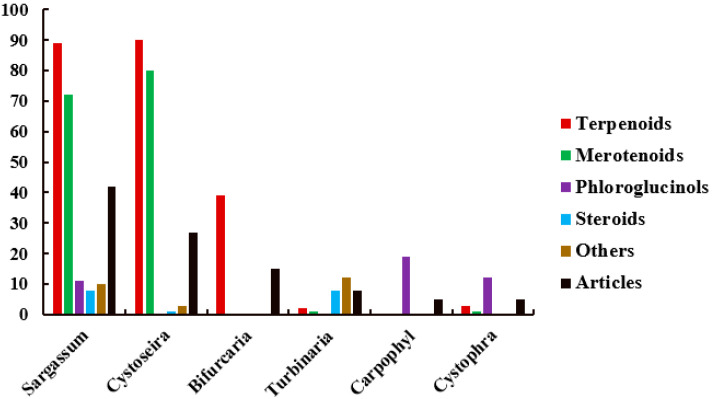
Numbers of compounds and publications from Sargassacean genus.

The majority of the secondary metabolites are meroterpenoids, diterpenoids, and phloroglucinols (Figure 22). *Sargassum* and *Cystoseira* are the most studied genera, reported by 42 and 27 articles, respectively, and are rich in meroterpenoids (Figure 23). *Bifurcaria*, investigated in 15 articles, is rich in linear diterpenoids, followed by *Turbinaria*, *Cystophora*, and *Carpophyllum*, which were discussed by eight, five, and five articles, respectively. Notably, the most productive species were *B. bifurcata* and *S. siliquastrum*, which have yielded 39 and 35 new compounds, respectively. They were followed by *C. usneoides*, *C. crinita* and *S. micracanthum*, which produced 22, 18, and 17 new compounds, respectively (Table 1).

Notably, from a chemical viewpoint, *B. bifurcata* is clearly distinguishable from other Sargassaceae species due to its extensive production of linear diterpenes. In contrast, the remaining species, with the exception of *C. crinita*, do not produce acyclic diterpenoids. Interestingly, the linear diterpenes yielded by *B. bifurcata* belong to mono-, dio-, and trioxygenated geranylgeraniol derivatives with the oxygenated function located at C-12, C-13, or C-10, depending on the specific sampling locations. 

Remarkably, a total of 134 compounds (Table 2), including 85 meroterpenoids, 16 diterpenoids, 2 triterpenoids, 5 phloroglucinols, 10 steroids, 3 macrolides, 3 pyran derivatives, 3 furanones, 3 spiroketals, 3 phenols, and one glycerol derivative, showed various biological activities, such as cytotoxic, antiprotozoal, antioxidant, antifouling, antiviral, antiglycation, antimicrobial, anti-Alzheimer’s disease, antidiabetic, antihypertensive, and antiphotoaging effects. Among them, 34 showed cytotoxicities against multiple cancer cell lines, including P388, A-549, L-1210, KB, HT-29, NSCLC-N6, MDA-MB-231, KA3IT, Colon26-L5, AGS, HT-1080, HEPG-2, HCT116, MCF-7, Caco-2, and HL-60. Structure-activity relationships indicated that the configuration of the double bond and positions/quantities/oxidation of hydroxyl groups played key roles in their cytotoxic activities. Additionally, 74 of them demonstrated potent radical-scavenging effects in the DPPH and ABTS assay, while 22 of them showed superior attenuation potential against cyclooxygenase-1/2 and 5-lipoxygenase, and TNF-α.

Therefore, Sargassacean algae are an important source of bioactive secondary metabolites. Given the great number of species of this family that remain chemically and pharmacologically underexplored, it is thus worthy to further investigate novel lead compounds from Sargassacean algae.

## Figures and Tables

**Table 1:** Chemical compounds studied in the Sargassaceae species in this review.

Species	Sampling Locations	Compounds and Types	Ref.
*Cystoseira schiffneri*	Chebba, Tunisia	**1** (monoterpenoid)	[27]
*C. crinita*	Catania, Sicily, Italy	**3**, **42**–**44**, **58** (sesquiterpenoid and diterpenoids)	[31,46]
	South coast of Sardinia, Italy	**70**, **71**, **73**–**78**, **80**, **81** (meroterpenoids)	[57]
	Toulon, France	**72**, **79**, **82** (meroterpenoids)	[58]
*C. myrica*	El-Zaafarana, Egypt	**63**–**66** (diterpenoids)	[52]
	Fayed, Egypt	**266** (steroids)	[117]
*C. abies*-*marina*	Mosteiros, Portugal	**83**, **84**, **87**, **88** (meroterpenoids)	[59]
	Punta del Hidalgo, Spain	**85**, **86**, **304** (meroterpenoids, ketone)	[60]
*C. amentacea* var. *stricta*	Le Brusc, Toulon, France	**89**, **166** (meroterpenoids)	[62]
*C. baccata*	El Jadida, Morocco	**90**, **91**, **167**–**173** (meroterpenoids)	[63,88]
*Cystoseira* sp.	Montaña Clara Island, Spain	**92**, **93**, **174**–**176** (meroterpenoids)	[64]
*C. balearica*	Portopalo, Sicily, Italy	**94** (meroterpenoid)	[66]
*C. stricta* var. *amentacea*	Castelluccio, Syracuse, Sicily, Italy	**95**, **96**, **104**–**107** (meroterpenoids)	[67,71]
*C. stricta*	Acicastello, Catania, Sicily, Italy	**97**–**100**, **108**, **109** (meroterpenoids)	[67,68,72]
	Portopalo, Sicily, Italy	**103** (meroterpenoid)	[70]
*C. stricta* var. *spicata*	near Cava d’Aliga, Italy	**101**, **102** (meroterpenoids)	[69]
*C. tamariscifolia*	Mediterranean Sea, Algeria	**110**–**113**, **177** (meroterpenoids)	[73]
*C. usneoides*	Mediterranean coast, Morocco	**114**–**119** (meroterpenoids)	[74]
	Tarifa, Spain	**120**–**131** (meroterpenoids)	[75]
	Sesimbra and Cabo Espichel, Portugal	**132**–**135** (meroterpenoids)	[76,77]
*C. sauvageuana*	Aci Castello, Sicily, Italy	**136**, **137** (meroterpenoids)	[78]
*C. barbata*	Salses, France	**306**, **307** (lipids)	[136]
*Sargassum naozhouense*	Leizhou Peninsula, China	**2**, **305** (monoterpenoid and amide)	[28,135]
*S. hemiphyllum*	Heda Coast, Izu Peninsula, Japan	**4**–**6** (norditerpenoids)	[32]
*S. micracanthum*	Kominato, Chiba, Japan	**7**–**14** (norditerpenoids)	[33]
	Coast of Gosa, Japan	**15**, **16** (norditerpenoids)	[34]
	Coast of Toyama Bay, Japan	**144**–**147**, **179**, **180** (meroterpenoids)	[82,91]
	Wando County, Korea	**227** (phloroglucinol)	[106]
*S. ilicifolium*	Gulf of Manner, India	**67** (diterpenoid)	[53]
*S. fallax*	Governor Reef near Indented Head, Port Phillip Bay, Australia	**139**, **140**, **178** (meroterpenoids)	[80]
*S. macrocarpum*	Coast of Tsukumowan, Japan	**141**–**143** (meroterpenoids)	[81]
*S. paradoxum*	Governor Reef near Indented Head, Australia	**148**–**151** (meroterpenoids)	[83]
*S. sagamianum*	Manazuru, Japan	**152**–**154**, **181**, **182** (meroterpenoids)	[84]
	Jeju Island, South Korea	**297** (glyceride)	[131]
*S. sagamianum* var. *yezoense*	Oshoro Bay, Japan	**155**, **156** (meroterpenoids)	[85]
*S. siliquastrum*	Jeju Island, Korea	**157**, **158**, **215**–**223**, **184**–**206** (meroterpenoids)	[86,93,94,95,96]
	Seashore of Pusan, Korea	**183** (meroterpenoids)	[92]
*S. tortile*	Awa-Kominato, Chiba, Japan	**159**–**165** (meroterpenoids)	[87]
	Tanabe Bay, Japan	**210**–**213** (meroterpenoids)	[99]
	Wakasa Bay, Japan	**214** (meroterpenoid)	[100,101]
*S. thun(m)bergii*	Coast of Busan, Korea	**208**, **209** (meroterpenoids)	[98]
	Muroran, Japan	**273** (steroid)	[122]
	Nanji Island, East China Sea, China	**274** (steroid)	[123]
	West Sea, Korea	**298**, **299** (glycolipids)	[132]
	Weihai, Shandong, China	**300**, **301** (resorcinols)	[133]
*S. nigrifoloides*	Nanji Island, Zhejiang, China	**224**–**226** (phloroglucinols)	[105]
*S. spinuligerum*	Wangaparoa Island, New Zealand	**228**, **235**–**239** (phloroglucinols)	[107,109]
	Auckland Harbour, New Zealand	**245**, **249** (phlorotannins)	[111]
*S. patens*	Coast of Noto Peninsula, Japan	**265** (phlorotannins)	[116]
*S. asperifolium*	Hurghada, Egypt	**267** (steroid)	[118]
*S. carpophyllum*	South China Sea, Beihai, China	**268**, **269** (steroids)	[119]
*S. fusiforme*	Anhui Bozhou Xiancheng Pharmaceutical Limited Company, China	**270**, **271** (steroids)	[120]
*S. polycystcum*	Weizhou Island, Beihai, China	**272** (steroid)	[121]
*S. parvivesiculosum*	Sanya, Hainan, China	**295**, **296** (glycerols)	[130]
*S. cinereum*	Red Sea, Hurghada, Egypt	**302**, **303** (aryl cresols)	[134]
*Cystophora moniliformis*	Port Phillip Bay, Victoria, Australia	**17**–**19** (norditerpenoids)	[35]
*C. harveyi*	East of Cape Leeuwin Lighthouse, Australia	**138** (meroterpenoid)	[79]
*C. torulosa*	Whangaparoa, New Zealand	**238**–**251** (phlorotannins)	[109,110,111]
*Bifurcaria bifurcata*	Atlantic coasts of Morocco	**20**–**22**, **24**–**26**, **60**, **62** (linear diterpenoids)	[36,37,39,51]
	Oualidia, Morocco	**23**, **27**, **59**, **61** (linear diterpenoids)	[38,40]
	Roscoff, Brittany, France	**28**, **29**, **50**–**57** (linear diterpenoids)	[41,42,43,44,45,46,47,48,49,50]
	Kilkee, County Clare of Ireland	**30**–**39**, **45** (linear diterpenoids)	[42,43,44]
	Quiberon, Brittany, France	**40**, **41**, **46**–**48** (linear diterpenoids)	[45]
	Near Piriac, France	**49** (linear diterpenoid)	[47]
*Turbinaria conoides*	Gulf of Manner, India	**207**, **283**–**288** (meroterpenoid, macrolides, and pyranoids)	[97,126,127]
	Salin Munthal, Gulf of Mannar, India	**275**, **276** (steroids)	[124]
	Kenting, Taiwan, China	**277**–**282** (steroids)	[125]
*T. ornata*	Indian peninsular, India	**289**–**291** (furanones)	[128]
	Gulf of Manner, India	**292**–**294** (spiroketals)	[129]
*T. decurrens*	Mandapam region, India	**68**, **69** (triterpenes)	[54]
*Carpophyllum maschalocarpum*	Torbay, north of Auckland, New Zealand	**229**–**234**, **252**, **253**, **257**–**261** (phlorotannins)	[108,112,114]
*C. angustifolium*	Panetiki Island, Cape Rodney, New Zealand	**254**–**256**, **262**–**264** (phlorotannins)	[113,115]

**Table 2:** Bioactive compounds reported from Sargassaceae species in this review.

Activity Class	Compounds	Biological Activities	Ref.
Cytotoxicity	**4**–**6**	against P388, IC_50_: 5.1, 2.2, and 50 μg/mL	[32]
	**132**, **133**	against P388, IC_50_: 0.8 and 1.5 μg/mL	[76]
		against A-549, IC_50_: 1.25 and 1.4 μg/mL	[76]
	**134**, **135**	against P-388, IC_50_: 3.2 and 6.8 μg/mL	[77]
		against L-1210, inhibition rate: 50–100%, 10–20 μg/mL	[77]
		against A-549, inhibition rate: 50–70%, 20 μg/mL	[77]
	**139**, **140**, **178**	against P388, IC_50_ > 27–29 μM	[80]
	**210**–**213**	against P388, ED_50_: 20.8, 14.0, 16.8 and 5.7 μg/mL	[99]
	**279**–**282**	against P-388, ED_50_: 0.6, 0.8, 0.9 and 0.4 μg/mL	[125]
		against KB, ED_50_: 5.9, 4.0, 4.6 and 1.8 μg/mL	[125]
		against A-549, ED_50_: 3.1, 2.5, 2.3 and 1.8 μg/mL	[125]
		against HT-29, ED_50_: 0.4, 1.4, 1.2 and 1.7 μg/mL	[125]
	**307**	against P388 in mice in vivo at 40 mg/kg	[136]
	**21**, **22**	against NSCLC-N6, IC_50_: 12.3 and 9.5 μg/mL	[37]
	**31**	against MDA-MB-231, inhibition rate: 78.8%, 100 μg/mL	[43]
	**35**	against MDA-MB-231, IC_50_: 30.7 μg/mL	[44]
	**63**–**66**	against KA3IT, IC_50_: 10, 5, 5 and 5 μg/mL	[52]
	**83**, **84**, **87**	against Hela in Log and Lag phases, IC_50_: 17.3–25.0, 20.1–32.0 and 2.8–10.2 μg/mL	[59]
	**152**, **153**, **181**	against Hela S3, IC_50_: 10, 4.0 and 10 μg/mL	[84]
	**144**–**146**	against Colon 26-L5, IC_50_: 1.51, 17.5 and 1.69 μg/mL	[82]
	**204**	against AGS, HT-29 and HT-1080, IC_50_: 6.5, 3.4 and 13.9 μg/mL	[95]
	**266**	against HEPG-2 and HCT116, IC_50_: 2.96 and 12.38 μM	[117]
	**302**	against HepG2, MCF-7 and Caco-2, IC_50_: 14.5, 17.6 and 18.2Μm	[134]
	**303**	against HepG2, MCF-7, and Caco-2, IC_50_: 13.1, 12.7 and 11.2 μM	[134]
	**268**	against HL-60, IC_50_: 2.96 μg/mL	[119]
		causing morphological abnormality of *Pyricularia oryzae* mycelia, MMDC: 63 μg/mL	[119]
	**269**	causing morphological abnormality of *P. oryzae* mycelia, MMDC: 250 μg/mL	[119]
	**28**, **62**, **89**	against *Paracentrotus lividus*, ED_50_: 12, 4 and 12 μg/mL	[41,51,62]
Anti-inflammatory	**67**	inhibit COX-1/2 and 5-LOX, IC_50_: 3.52, 2.47 and 4.70 mM	[53]
	**68**, **69**	inhibit COX-1, IC_50_: 21.62 and 22.02 μM	[54]
		inhibit COX-2, IC_50_: 15.51 and 13.98 μM	[54]
		inhibit 5-LOX, IC_50_: 3.92 and 3.02 μM	[54]
	**207**	inhibit COX-2 and 5-LOX, IC_50_: 1.47 and 3.70 μM	[97]
	**283**–**288**	inhibit COX-1, IC_50_: 3.13, 3.19, 3.35, 4.06, 5.11 and 5.23 mM	[126,127]
		inhibit COX-2, IC_50_: 1.75, 1.93, 1.99, 2.15, 2.93 and 3.27 mM	[126,127]
		inhibit 5-LOX, IC_50_: 4.24, 4.88, 5.07, 2.41, 2.99 and 3.22 mM	[126,127]
	**297**	inhibit COX-2 and sPLA2-IIA, inhibition rate: 35.6%, 50 μM; 26.1%, 10 μM	[131]
	**114**, **115**, **117**	TNF-α inhibition, inhibition rate: 11–33%, 6–10 μM	[74]
	**120**	TNF-α inhibition, inhibition rate: 81%, 10 μM	[75]
	**121**, **123**, **127**, **129**, **130**	TNF-α inhibition, inhibition rate: 21–35%, 8–10 μM	[75]
	**125**	TNF-α inhibition, inhibition rate: 79%, 8 μM	[75]
	**128**	59% inhibition against TNF-α at 5 μM	[75]
Antioxidant	**67**	scavenge DPPH and ABTS^+^ radicals, IC_50_: 1.26 and 1.38 mM	[53]
	**70**, **71**, **73**–**78**, **80**, **81**	scavenge DPPH radicals, scavenging rate: 29.0–96.7%, 164–230 μM	[57]
	**87**, **88**	scavenge DPPH radicals, scavenging rate: 29–30%, 500 μg/mL	[59]
	**114**–**117**	scavenge ABTS˙^+^ radicals, EC_50_: 22.5–55.9 μM	[72]
	**120**–**125**, **127**–**130**	scavenge ABTS˙^+^ radicals, EC_50_: 14.81–32.41 μM	[75]
	**144**–**146**	inhibition lipid peroxidation, IC_50_: 0.95–44.3 μg/mL	[82]
		scavenge DPPH radicals, IC_50_: 3.00– 52.6 μg/mL	[82]
	**157**	scavenge DPPH radicals, RC_50_: 0.24 μg/mL	[86]
	**183**	scavenge DPPH radicals, scavenging rate: 96.07%, 0.5 mg/mL	[92]
	**187**–**202**	scavenge DPPH radicals, scavenging rate: 87–91%, 100 μg/mL	[94]
	**205**, **206**	scavenge DPPH radicals, EC_50_: 31.1–57.1 mM	[96]
		scavenge ABTS^+^ radicals, EC_50_: 15.8–28.1 μM	[96]
	**207**	scavenge DPPH and ABTS^+^ radicals, IC_50_: 24.25 and 24.32 μM	[97]
	**208**, **209**	scavenge DPPH radicals, EC_50_: 30 and 31 μg/mL	[98]
		scavenge authentic/induced ONOO-, scavenging rate: 60/98.6%, 57.1/90.6%	[98]
	**215**–**219**	scavenge DPPH radicals, RC_50_: 11.72–23.23 μg/mL	[86]
	**220**–**223**	scavenge DPPH radicals, RC_50_: 0.10–0.33 μg/mL	[86]
	**227**	scavenge ABTS+ radicals, IC_50_: 47 μM	[106]
	**283**–**285**	scavenge DPPH radicals, IC_50_: 1.20, 1.35 and 1.54 mM	[126]
		scavenge ABTS^+^ radicals, IC_50_: 1.48, 1.54, and 1.81mM	[126]
	**286**–**288**	scavenge DPPH radicals, IC_50_: 0.54, 0.54 and 0.68 mg/mL	[127]
		scavenge ABTS^+^ radicals, IC_50_: 0.58, 0.58 and 0.76 mg/mL	[127]
	**289**–**291**	scavenge DPPH radicals, IC_50_: 1.16, 1.05 and 1.21 mM	[128]
		scavenge ABTS^+^ radicals, IC_50_: 1.38, 1.24 and 1.41 mM	[128]
	**292**–**294**	scavenge DPPH radicals, IC_50_: 1.14, 1.25 and 1.42 mM	[129]
		scavenge ABTS^+^ radicals, IC_50_: 1.28, 1.34 and 1.71 mM	[129]
	**184**–**186**	reduce ROS formation in HT 1080 cells by over 67.2% at 5 μg/mL	[93]
		inhibit lipid peroxidation induced by H_2_O_2_	[93]
		increase GSH levels in HT1080 cells at 5 μg/mL	[93]
Antifouling	**110**–**113**, **177**	against *Pseudoalteromonas elyakovii*, *Vibrio aesturianus*, *Polaribacter irgensii*, *Halosphaeriopsis mediosetigera*, *Asteromyces cruciatus*, and *Lulworthia uniseptate*, MIC: 0.1–10 μg/mL	[73]
		against *Exanthemachrysis gayraliae*, *Cylindrotheca closterium*, *Pleurochrysis roscoffensis*, *Ulva intestinalis*, and *Undaria pinnatifida*, MIC: 0.1–10 μg/mL	[73]
	**168**	against *Sargassum muticum* and phenoloxidase, IC_50_: 2.5 and 1 μg/mL	[63]
	**169**	against *S. muticum*, *U. intestinalis*, phenoloxidase, and *E. gayraliae*, IC_50_: 1 μg/mL	[63]
	**171**	against *U. intestinalis* and phenoloxidase, IC_50_: 2.5 and 2.5 μg/mL	[63]
Antimicrobial	**149**–**151**	against *Streptococcus pyogenes* (345/1), zones of inhibition: 1–3 mm, 1 mg/mL	[83]
	**152**, **153**, **181**	against *Bacillus subtilis* and *Staphylococcus aureus*, inhibition rate: ca. 30 and 80%	[84]
	**157**	slight inhibition against isocitrate lyase from *S. aureus*	[86]
	**158**, **215**–**223**	weak inhibition AGAINST sortase A from *Candida albicans*	[86]
	**275**	against *Staphylococcus aureus*, *S. epidermidis*, *Escherichia coli* and *Pseudomonas aeruginosa*, MIC: 32–128 μg/mL	[124]
		against *Candida albicans* and *Aspergillus niger*, MIC: 16 μg/mL	[124]
	**276**	against *S. aureus*, *S. epidermidis*, *E. coli* and *P. aeruginosa*, MIC: 32–128 μg/mL	[124]
		against *C. albicans* and *A. niger*, MIC: 4 and 2 μg/mL	[124]
Anti-Alzheimer’s disease	**193**, **201**	butylcholine esterase inhibition, inhibition rates: 82.7 or 80%	[94]
	**224**–**226**	against CDK5, IC_50_: 12, 18 and 17 μM	[105]
		against GSK3β, IC_50_: 1.6, 1.1 and 1.8 μM	[105]
Antidiabetic	**265**	against α-amylase and α-glucosidase with IC_50_ values of 3.2 and 25.4–114 μg/mL, respectively	[116]
	**274**	PTP1B inhibition, IC_50_: 2.24 mM	[123]
	**283**–**285**	PTP-1B inhibition, IC_50_: 1.39, 2.33 and 3.13 mM	[126]
	**289**–**291**	PTP-1B inhibition, IC_50_: 2.58, 2.42 and 2.77 mM	[128]
		α-amylase inhibition, IC_50_: 0.39, 0.31 and 0.48 mM	[128]
		α-glucosidase inhibition, IC_50_: 0.34, 0.27 and 0.44 mM	[128]
Antihypertensive	**283**–**285**	ACE-I inhibition, IC_50_: 1.23, 1.89 and 2.23 mM	[126]
	**292**–**294**	ACE-I inhibition, IC_50_: 4.55, 4.72 and 4.86 mM	[129]
Antiprotozoal	**30**	against *Plasmodium falciparum*, IC_50_: 0.65 μg/mL	[42]
Antiviral	**132**–**135**	against CV-1, IC_50_: 4.0, 1.0, 3.6 and 4.0 μg/mL	[76,77]
		against BHK, IC_50_: 6.2, 1.1, 3.7 and 6.2 μg/mL	[76,77]
Antiglycation	**141**–**143**	AGEs inhibition, IC_50_: 2.1, 2.6 and 1.0 mM	[81]
Antiphotoaging	**172**, **173**	photodamage attenuation effect, cell viability value: 82.6–95.1%, 5–20 μg/mL	[90]

## Data Availability

The data presented in this study are available.

## References

[1] Kumari P., Kumar M., Gupta V., Reddy C.R.K., Jha B. (2010). Tropical marine macroalgae as potential sources of nutritionally important PUFAs. Food Chem..

[2] Anis M., Ahmed S., Hasan M.M. (2017). Algae as nutrition, medicine and cosmetic: The forgotten history, present status and future trends. World J. Pharm. Sci..

[3] Yende S.R., Harle U.N., Chaugule B.B. (2014). Therapeutic potential and health benefits of *Sargassum* species. Phcog. Rev..

[4] Muñoz J., Culioli G., Köck M. (2013). Linear Diterpenes from the Marine Brown Alga *Bifurcaria bifurcata*: A chemical perspective. Phytochem. Rev..

[5] Remya R.R., Samrot A.V., Kumar S.S., Mohanavel V., Karthick A., Chinnaiyan V.K., Umapathy D., Muhibbullah M. (2022). Bioactive potential of brown algae. Adsorpt. Sci. Technol..

[6] Arrieche D., Carrasco H., Olea A.F., Espinoza L., San-Martín A. (2022). Secondary metabolites isolated from Chilean Marine Algae: A Review. Mar. Drugs.

[7] Máximo P., Ferreira L.M., Branco P., Lima P., Lourenço A. (2018). Secondary metabolites and biological activity of invasive macroalgae of Southern Europe. Mar. Drugs.

[8] Peng Y., Hu J., Yang B., Lin X.P., Zhou X.F., Yang X.W., Liu Y.H., Tiwari B.K., Troy D.J. (2015). Chemical Composition of Seaweed. Seaweed Sustainability: Food and Non-Food Applications.

[9] Balboa E.M., Conde E., Moure A., Falqué E., Domínguez H. (2013). *In vitro* antioxidant properties of crude extracts and compounds from brown algae. Food Chem..

[10] Generalić Mekinić I., Skroza D., Šimat V., Hamed I., Čagalj M., Popović Perković Z. (2019). Phenolic content of brown algae (*Pheophyceae*) species: Extraction, identification, and quantification. Biomolecules.

[11] Huang B., Ding L., Luan R., Sun Z. (2015). New classification system of marine brown algae of China. Guangxi Sci..

[12] Carroll A.R., Copp B.R., Davis R.A., Keyzers R.A., Prinsep M.R. (2022). Marine natural products. Nat. Prod. Rep..

[13] Blunt J.W., Carroll A.R., Copp B.R., Davis R.A., Keyzers R.A., Prinsep M.R. (2017). Marine natural products. Nat. Prod. Rep..

[14] Draisma S.G.A., Ballesteros E., Rousseau F., Thibaut T. (2010). DNA sequence data demonstrate the polyphyly of the genus *Cystoseira* and other *Sargassaceae genera* (Phaeophyceae). J. Phycol..

[15] Rousseau F., de Reviers B. (1999). Phylogenetic relationships within the Fucales (Phaeophyceae) based on combined partial SSU + LSU rDNA sequence data. Eur. J. Phycol..

[16] Guiry M.D., Guiry G.M. AlgaeBase. World-Wide Electronic Publication, National University of Ireland, Galway. http://www.algaebase.org.

[17] Liu L., Heinrich M., Myers S., Dworjanyn S.A. (2012). Towards a better understanding of medicinal uses of the brown seaweed *Sargassum* in Traditional Chinese Medicine: A phytochemical and pharmacological review. J. Ethnopharmacol..

[18] de Sousa C.B., Gangadhar K.N., Macridachis J., Pavão M., Morais T.R., Campino L., Varela J., Lago J.H.G. (2017). *Cystoseira* algae (Fucaceae): Update on their chemical entities and biological activities. Tetrahedron: Asymmetry.

[19] Catarino M.D., Pires S.M.G., Silva S., Costa F., Braga S.S., Pinto D.C.G.A., Silva A.M.S., Cardoso S.M. (2022). Overview of phlorotannins’ constituents in *Fucales*. Mar. Drugs.

[20] Valls R., Piovetti L. (1995). The chemistry of the *Cystoseiraceae* (Fucales: Pheophyceae): Chemotaxonomic relationships. Biochem. Syst. Ecol..

[21] Gouveia V., Seca A.M.L., Barreto M.C., Pinto D.C.G.A. (2013). Di- and sesquiterpenoids from *Cystoseira* genus: Structure, intra-molecular transformations and biological activity. Mini-Rev. Med. Chem..

[22] Chen Z., Liu H.B. (2012). Recent Advance on the Chemistry and Bioactivity of Genus *Sargassum*. Chin. J. Mar. Drugs.

[23] Rushdi M.I., Abdel-Rahman I.A.M., Saber H., Attia E.Z., Abdelraheem W.M., Madkour H.A., Hassan H.M., Elmaidomy A.H. (2020). Pharmacological and natural products diversity of the brown algae genus *Sargassum*. RSC Adv..

[24] Rushdi M.I., Abdel-Rahman I.A.M., Saber H., Attia E.Z., Abdelraheem W.M., Madkour H.A., Abdelmohsen U.R. (2021). The genus *Turbinaria*: Chemical and pharmacological diversity. Nat. Prod. Res..

[25] Xu R.S., Ye Y., Zhao W.M. (2004). Natural Products Chemistry.

[26] Roberts S.C. (2007). Production and engineering of terpenoids in plant cell culture. Nat. Chem. Biol..

[27] Salem A.B., Di Giuseppe G., Anesi A., Hammami S., Mighri Z., Guella G. (2017). Natural products among brown algae: The case of *Cystoseira schiffneri* Hamel (Sargassaceae, Phaeophyceae). Chem. Biodivers..

[28] Peng Y., Huang R.M., Lin X.P., Liu Y.H. (2018). Norisoprenoids from the brown alga *Sargassum naozhouense* Tseng et Lu. Molecules.

[29] Smith A.B., Branca S.J., Pilla N.N., Guaciaro M.A. (1982). Stereocontrolled total synthesis of (±)-pentenomycins. I-III, their epimers, and dehydropentenomycin I. J. Org. Chem..

[30] Kimura J., Maki N. (2002). New loliolide derivatives from the brown alga *Undaria pinnatifa*. J. Nat. Prod..

[31] Fattorusso E., Magno S., Mayol L., Santacroce C., Sica D., Amico V., Oriente G., Piattelli M., Tringal C. (1976). Oxocrinol and Crinitol, novel linear terpenoids from the brown alga *Cystoseira crinita*. Tetrahedron Lett..

[32] Takada N., Watanabe R., Suenaga K., Yamada K., Uemura D. (2001). Isolation and structures of hedaols A, B, and C, new bisnorditerpenes from a Japanese brown alga. J. Nat. Prod..

[33] Kusumi T., Ishitsuka M., Nomura Y., Konno T., Kakisawa H. (1979). New farnesylacetone derivatives from *Sargassum micracanthum*. Chem. Lett..

[34] Shizuri Y., Matsukawa S., Ojika M., Yamada K. (1982). Two new farnesylacetone derivatives from the brown alga *Sargassum micracanthum*. Phytochemistry.

[35] Reddy P., Urban S. (2008). Linear and cyclic C18 terpenoids from the Southern Australian marine brown alga *Cystophora moniliformis*. J. Nat. Prod..

[36] Valls R., Banaigs B., Francisco C., Codomier L., Cave A. (1986). An acyclic diterpene from the brown alga *Bifurcaria bifurcata*. Phytochemistry.

[37] Culioli G., Ortalo-Magné A., Daoudi M., Thomas-Guyon H., Valls R., Piovetti L. (2004). Trihydroxylated linear diterpenes from the brown alga *Bifurcaria bifurcata*. Phytochemistry.

[38] El Hattab M., Ben Mesaoud M., Daoudi M., Ortalo-Magné A., Culioli G., Valls R., Piovetti L. (2008). Trihydroxylated linear diterpenes from the brown alga *Bifurcaria bifurcata* (Fucales, Phaeophyta). Biochem. Syst. Ecol..

[39] Culioli G., Daoudi M., Ortalo-Magné A., Valls R., Piovetti L. (2001). (S)-12-hydroxygeranylgeraniol-derived diterpenes from the brown alga *Bifurcaria bifurcata*. Phytochemistry.

[40] Semmak L., Zerzouf A., Valls R., Banaigs B., Jeanty G., Francisco C. (1988). Acyclic diterpenes from *Bifurcaria bifurcata*. Phytochemistry.

[41] Valls R., Piovetti L., Banaigs B., Archavlis A., Pellegrini M. (1995). (S)-13-hydroxygeranylgeraniol-derived furanoditerpenes from *Bifurcaria bifurcata*. Phytochemistry.

[42] Smyrniotopoulos V., Merten C., Kaiser M., Tasdemir D. (2017). Bifurcatriol, a new antiprotozoal acyclic diterpene from the brown alga *Bifurcaria bifurcata*. Mar. Drugs.

[43] Smyrniotopoulos V., Firsova D., Fearnhead H., Grauso L. (2021). Density functional theory (DFT)-aided structure elucidation of linear diterpenes from the Irish brown seaweed *Bifurcaria bifurcata*. Mar. Drugs.

[44] Smyrniotopoulos V., Merten C., Firsova D., Fearnhead H., Tasdemir D. (2020). Oxygenated acyclic diterpenes with anticancer activity from the Irish brown seaweed *Bifurcaria bifurcata*. Mar. Drugs.

[45] Ortalo-Magné A., Culioli G., Valls R., Pucci B., Piovetti L. (2005). Polar acyclic diterpenoids from *Bifurcaria bifurcata*. Phytochemistry.

[46] Amico V., Oriente G., Piattelli M., Ruberto G., Tringali C. (1981). Novel acyclic diterpenes from the brown alga *Cystoseira crinita*. Phytochemistry.

[47] Combaut G., Piovetti L. (1983). A novel acyclic diterpene from the brown alga *Bifurcaria bifurcata*. Phytochemistry.

[48] Göthel Q., Muñoz J., Köck M. (2012). Formyleleganolone and bibifuran, two metabolites from the brown alga *Bifurcaria bifurcata*. Phytochem. Lett..

[49] Göthel Q., Lichte E., Köck M. (2012). Further eleganolone-derived diterpenes from the brown alga *Bifurcaria bifurcata*. Tetrahedron Lett..

[50] Hougaard L., Anthoni U., Christophersen C. (1991). Two new diterpenoid dihydroxy-γ-butyrolactones from *Bifurcaria bifurcata* (Cystoseiraceae). Tetrahedron Lett..

[51] Valls R., Banaigs B., Piovetti L., Archavlis A., Artaud J. (1993). Linear diterpene with antimitotic activity from the brown alga *Bifurcaria bifurcata*. Phytochemistry.

[52] Ayyad S.E.N., Abdel-Halim O.B., Shier W.T., Hoye T.R. (2003). Cytotoxic hydroazulene diterpenes from the brown alga *Cystoseira myrica*. Z. Naturforsch. C.

[53] Dhara S., Chakraborty K. (2021). Anti-inflammatory xenicane-type diterpenoid from the intertidal brown seaweed *Sargassum ilicifolium*. Nat. Prod. Res..

[54] Thambi A., Chakraborty K. (2023). Anti-inflammatory decurrencyclics A-B, two undescribed nor-dammarane triterpenes from triangular sea bell *Turbinaria decurrens*. Nat. Prod. Res..

[55] Joung E.-J., Gwon W.-G., Shin T., Jung B.-M., Choi J., Kim H.-R. (2017). Anti-inflammatory action of the ethanolic extract from *Sargassum serratifolium* on lipopolysaccharide-stimulated mouse peritoneal macrophages and identification of active components. J. Appl. Phycol..

[56] Azam M.S., Choi J., Lee M.-S., Kim H.-R. (2017). Hypopigmenting effects of brown algae-derived phytochemicals: A review on molecular mechanisms. Mar. Drugs.

[57] Fisch K.M., Böhm V., Wright A.D., König G.M. (2003). Antioxidative meroterpenoids from the brown alga *Cystoseira crinita*. J. Nat. Prod..

[58] Praud A., Valls R., Piovetti L., Banaigs B., BenaΪm J.-Y. (1995). Meroditerpenes from the brown alga *Cystoseira crinita* off the French Mediterranean coast. Phytochemistry.

[59] Gouveia V.L.M., Seca A.M.L., Barreto M.C., Neto A.I., Kijjoa A., Silva A.M.S. (2013). Cytotoxic meroterpenoids from the macroalga *Cystoseira abies*-*marina*. Phytochem. Lett..

[60] Fernández J.J., Navarro G., Norte M. (1998). Novel metabolites from the brown alga *Cystoseira abies-marina*. Nat. Prod. Lett..

[61] García P.A., Hernández Á.P., San Feliciano A., Castro M.Á. (2018). Bioactive prenyl- and terpenyl-quinones/hydroquinones of marine origin. Mar. Drugs.

[62] Mesguiche V., Valls R., Piovetti L., Banaigs B. (1997). Meroditerpenes from *Cystoseira amentacea* var. *stricta* collected off the Mediterranean coasts. Phytochemistry.

[63] Mokrini R., Mesaoud M.B., Daoudi M., Hellio C., Maréchal J.-P., El Hattab M., Ortalo-Magné A., Piovetti L., Culioli G. (2008). Meroditerpenoids and derivatives from the brown alga *Cystoseira baccata* and their antifouling properties. J. Nat. Prod..

[64] Navarro G., Fernández J.J., Norte M. (2004). Novel meroditerpenes from the brown alga *Cystoseira* sp.. J. Nat. Prod..

[65] Amico V. (1995). Marine brown algae of family Cystoseiraceae: Chemistry and chemotaxonomy. Phytochemistry.

[66] Amico V., Cunsolo F., Piattelli M., Ruberto G., Fronczek F.R. (1984). Balearone, a metabolite of the brown alga *Cystoseira balearica*. Tetrahedron.

[67] Amico V., Piattelli M., Neri P., Ruberto G., Mayol L. (1986). Novel metabolites from the marine genus *cystoseira*-application of two-dimensional ^1^H-^13^C correlation to the structure elucidation. Tetrahedron.

[68] Amico V., Cunsolo F., Piattelli M., Ruberto G. (1987). Prenylated *O*-methyltoluquinols from *Cystoseira stricta*. Phytochemistry.

[69] Amico V., Oriente G., Piattelli M., Ruberto G., Tringali C. (1982). A quinone-hydroquinone couple from the brown alga *Cystoseira stricta*. Phytochemistry.

[70] Amico V., Cunsolo F., Piattelli M., Ruberto G., Mayol L. (1987). Strictaketal, a new tetraprenyltoluquinol with a heterotetracyclic diterpene moiety from the brown alga *Cystoseira stricta*. J. Nat. Prod..

[71] Amico V., Oriente G., Neri P., Piattelli M., Ruberto G. (1987). Tetraprenyltoluquinols from the brown alga *Cystoseira stricta*. Phytochemistry.

[72] Amico V., Piattelli M., Cunsolo F., Neri P., Ruberto G. (1989). Two epimeric, irregular diterpenoid toluquinols from the brown alga *Cystoseira stricta*. J. Nat. Prod..

[73] El Hattab M., Genta-Jouve G., Bouzidi N., Ortalo-Magné A., Hellio C., Maréchal J.-P., Piovetti L., Thomas O.P., Culioli G. (2015). Cystophloroketals A-E, unusual phloroglucinol-meroterpenoid hybrids from the brown alga *Cystoseira tamariscifolia*. J. Nat. Prod..

[74] de los Reyes C., Zbakh H., Motilva V., Zubía E. (2013). Antioxidant and anti-inflammatory meroterpenoids from the brown alga *Cystoseira usneoides*. J. Nat. Prod..

[75] de los Reyes C., Ortega M.J., Zbakh H., Motilva V., Zubía E. (2016). *Cystoseira usneoides*: A brown alga rich in antioxidant and anti-inflammatory meroditerpenoids. J. Nat. Prod..

[76] Urones J.G., Basabe P., Marcos I.S., Pineda J., Lithgow A.M., Moro R.F., Palma F.M.S.B., Araújo M.E.M., Gravalos M.D.G. (1992). Meroterpenes from *Cystoseira usneoides*. Phytochemistry.

[77] Urones J.G., Araujo M.E.M., Palma F.M.S.B., Basabe P., Marcos I.S., Moro R.F., Lithgow A.M., Pineda J. (1992). Meroterpenes from *Cystoseira usneoides* II. Phytochemistry.

[78] Amico V., Cunsolo F., Piattelli M., Ruberto G. (1985). Acyclic tetraprenyltoluquinols from *Cystoseira sauvageuana* and their possible role as biogenetic precursors of the cyclic *Cystoseira* metabolites. Phytochemistry.

[79] Laird D.W., Poole R., Wikström M., van Altena I.A. (2007). Pycnanthuquinone C, an unusual 6,6,5-tricyclic geranyltoluquinone from the Western Australian brown alga *Cystophora harveyi*. J. Nat. Prod..

[80] Reddy P., Urban S. (2009). Meroditerpenoids from the southern Australian marine brown alga *Sargassum fallax*. Phytochemistry.

[81] Niwa H., Kurimoto S., Kubota T., Sekiguchi M. (2021). Macrocarquinoids A–C, new meroterpenoids from *Sargassum macrocarpum*. J. Nat. Med..

[82] Mori J., Iwashima M., Wakasugi H., Saito H., Matsunaga T., Ogasawara M., Takahashi S., Suzuki H., Hayashi T. (2005). New plastoquinones isolated from the brown alga, *Sargassum micracanthum*. Chem. Pharm. Bull..

[83] Brkljača R., Urban S. (2015). Chemical profiling (HPLC-NMR & HPLC-MS), isolation, and identification of bioactive meroditerpenoids from the southern Australian marine brown alga *Sargassum paradoxum*. Mar. Drugs.

[84] Horie S., Tsutsumi S., Takada Y., Kimura J. (2008). Antibacterial quinone metabolites from the brown alga, *Sargassum sagamianum*. Bull. Chem. Soc. Jpn..

[85] Segawa M., Shirahama H. (1987). New plastoquinones from the brown alga *Sargassum sagamianum* var. *yezoense*. Chem. Lett..

[86] Jung M., Jang K.H., Kim B., Lee B.H., Choi B.W., Oh K.-B., Shin H. (2008). Meroditerpenoids from the brown alga *Sargassum siliquastrum*. J. Nat. Prod..

[87] Ishitsuka M., Kusumi T., Nomura Y., Konno T., Kakisawa H. (1979). New geranylgeranylbenzoquinone derivatives from *Sargassum tortile*. Chem. Lett..

[88] Valls R., Piovetti L., Banaigs B., Praud A. (1993). Secondary metabolites from Morocco brown algae of the genus *Cystoseira*. Phytochemistry.

[89] Fuentes-Monteverde J.C.C., Nath N., Forero A.M., Balboa E.M., Navarro-Vázquez A., Griesinger C., Jiménez C., Rodríguez J. (2022). Connection of isolated stereoclusters by combining ^13^C-RCSA, RDC, and J-based configurational analyses and structural revision of a tetraprenyltoluquinol chromane meroterpenoid from *Sargassum muticum*. Mar. Drugs.

[90] Balboa E.M., Li Y.-X., Ahn B.-N., Eom S.-H., Domínguez H., Jiménez C., Rodríguez J. (2015). Photodamage attenuation effect by a tetraprenyltoluquinol chromane meroterpenoid isolated from *Sargassum muticum*. J. Photochem. Photobiol. B..

[91] Iwashima M., Tako N., Hayakawa T., Matsunaga T., Mori J., Saito H. (2008). New chromane derivatives isolated from the brown alga, *Sargassum micracanthum*. Chem. Pharm. Bull..

[92] Cho S.H., Cho J.Y., Kang S.E., Hong Y.K., Ahn D.H. (2008). Antioxidant activity of mojabanchromanol, a novel chromene, isolated from brown alga *Sargassum siliquastrum*. J. Environ. Biol..

[93] Lee J.I., Seo Y. (2011). Chromanols from *Sargassum siliquastrum* and their antioxidant activity in HT1080 cells. Chem. Pharm. Bull..

[94] Jang K.H., Lee B.H., Choi B.W., Lee H.S., Shin J. (2005). Chromenes from the brown alga *Sargassum siliquastrum*. J. Nat. Prod..

[95] Lee J.-I., Park B.J., Kim H., Seo Y. (2014). Isolation of two new meroterpenoids from *Sargassum siliquastrum*. Bull. Korean. Chem. Soc..

[96] Kang H.-S., Kim J.-P. (2017). New chromene derivatives with radical scavenging activities from the brown alga *Sargassum siliquastrum*. J. Chem. Res..

[97] Dhara S., Chakraborty K. (2022). Novel furanyl-substituted isochromanyl class of anti-inflammatory turbinochromanone from brown seaweed *Turbinaria conoides*. Chem. Biodivers..

[98] Seo Y., Park K.E., Kim Y.A., Lee H.-J., Yoo J.-S., Ahn J.-W., Lee B.-J. (2006). Isolation of tetraprenyltoluquinols from the brown alga *Sargassum thunbergii*. Chem. Pharm. Bull..

[99] Numata A., Kanbara S., Takahashi C., Fujiki R., Yoneda M., Usami Y., Fujita E. (1992). A cytotoxic principle of the brown alga *Sargassum tortile* and structures of chromenes. Phytochemistry.

[100] Kikuchi T., Mori Y., Yokoi T., Nakazawa S., Kuroda H., Masada Y., Kitamura K., Umezaki I. (1975). Structure of sargatriol, a new isoprenoid chromenol from a marine alga: *Sargassum tortile*. Chem. Pharm. Bull..

[101] Kikuchi T., Mori Y., Yokoi T., Nakazawa S., Kuroda H., Masada Y., Kitamura K., Kuriyama K. (1983). Structure and absolute configuration of sargatriol, a new isoprenoid chromenol from a brown alga, *Sargassum tortile* C. Agardh. Chem. Pharm. Bull..

[102] Jung M., Jang K.H., Kim B., Lee B.H., Choi B.W., Oh K.-B., Shin H. (2009). Meroditerpenoids from the brown alga *Sargassum siliquastrum*. J. Nat. Prod..

[103] Gunathilake T., Akanbi T.O., Suleria H.A.R., Nalder T.D., Francis D.S., Barrow C.J. (2022). Seaweed phenolics as natural antioxidants, aquafeed additives, veterinary treatments and cross-linkers for microencapsulation. Mar. Drugs.

[104] Singh I.P., Bharate S.B. (2006). Phloroglucinol compounds of natural origin. Nat. Prod. Rep..

[105] Jiang C.-S., Wang Y.-Y., Song J.-T., Yu J.-H. (2018). Acylphloroglucinols as kinase inhibitors from *Sargassum nigrifoloides*. J. Asian Nat. Prod. Res..

[106] Kim C., Lee I.-K., Cho G.Y., Oh K.-H., Lim Y.W., Yun B.-S. (2012). Sargassumol, a novel antioxidant from the brown alga *Sargassum micracanthum*. J. Antibiot..

[107] Keusgen M., Falk M., Walter J.A., Glombitza K.-W. (1997). A phloroglucinol derivative from brown alga *Sargassum spinuligerum*. Phytochemistry.

[108] Glombitza K.-W., Li S.-M. (1991). Fucophlorethols from the brown alga *Carpophyllum maschalocarpum*. Phytochemistry.

[109] Glombitza K.-M., Keusgen M., Hauperich S. (1997). Fucophlorethols from the brown algae *Sargassum spinuligerum* and *Cystophora torulosa*. Phytochemistry.

[110] Glombitza K.-W., Hauperich S. (1997). Phlorotannins from the brown alga *Cystophora torulosa*. Phytochemistry.

[111] Glombitza K.-M., Hauperich S., Keusgen M. (1997). Phlorotannins from the brown algae *Cystophora torulosa* and *Sargassum spinuligerum*. Nat. Toxins.

[112] Glombitza K.-W., Li S.-M. (1991). Hydroxyphlorethols from the brown alga *Carpophyllum maschalocarpum*. Phytochemistry.

[113] Glombitza K.-M., Schmidt A. (1999). Trihydroxyphlorethols from the brown alga *Carpophyllum angustifolium*. Phytochemistry.

[114] Li S.-M., Glombitza K.-W. (1991). Carmalols and phlorethofuhalols from the brown alga *Carpophyllum maschalocarpum*. Phytochemistry.

[115] Glombitza K.-W., Schmidt A. (1999). Nonhalogenated and halogenated phlorotannins from the brown alga *Carpophyllum angustifolium*. J. Nat. Prod..

[116] Kawamura-Konishi Y., Watanabe N., Saito M., Nakajima N., Sakaki T., Katayama T., Enomoto T. (2012). Isolation of a new phlorotannin, a potent inhibitor of carbohydrate-hydrolyzing enzymes, from the brown alga *Sargassum patens*. J. Agric. Food Chem..

[117] Hamdy A.-H.A., Aboutabl E.A., Sameer S., Hussein A.A., Díaz-Marrero A.R., Darias J., Cueto M. (2009). 3-Keto-22-epi-28-nor-cathasterone, a brassinosteroid-related metabolite from *Cystoseira myrica*. Steroids.

[118] Ayyad S.-E.N., Sowellim S.Z.A., El-Hosini M.S., Abo-Atia A. (2003). The structural determination of a new steroidal metabolite from the brown alga *Sargassum asperifolium*. Z. Naturforsch. C..

[119] Tang H.-F., Yi Y.-H., Yao X.-S., Xu Q.-Z., Zhang S.-Y., Lin H.-W. (2002). Bioactive steroids from the brown alga *Sargassum carpophyllum*. J. Asian Nat. Prod. Res..

[120] Wang S.-Y., Xiang J., Huang X.-J., Wei X., Zhang Q., Du X.-H., Hu J.-S., Wang Y.-M., Zhang C.-X. (2020). Chemical constituents from *Sargassum fusiforme* (Harv.) Setch. Chem. Biodivers..

[121] Xu S.-H., Ding L.-S., Wang M.-K., Peng S.-L., Liao X. (2002). Studies on the chemical constituents of the algae *Sargassum polycystcum*. Chin. J. Org. Chem..

[122] Kobayashi M., Hasegawa A., Mitsuhashi H. (1985). Marine sterols. XV. Isolation of 24-vinyloxycholesta-5,23-dien-3*β*-ol from the brown alga *Sargassum thumbergii*. Chem. Pharm. Bull..

[123] He W.-F., Yao L.-G., Liu H.-L., Guo Y.-W. (2014). Thunberol, a new sterol from the Chinese brown alga *Sargassum thunbergii*. J. Asian Nat. Prod. Res..

[124] Kumar S.S., Kumar Y.K., Khan M.S.Y., Gupta V. (2010). New antifungal steroids from *Turbinaria conoides* (J.Agardh) Kutzing. Nat. Prod. Res..

[125] Sheu J.-H., Wang G.-H., Sung P.-J., Duh C.-Y. (1999). New cytotoxic oxygenated fucosterols from the brown alga *Turbinaria conoides*. J. Nat. Prod..

[126] Chakraborty K., Dhara S. (2021). Conoidecyclics A-C from marine macroagla *Turbinaria conoides*: Newly described natural macrolides with prospective bioactive properties. Phytochemistry.

[127] Chakraborty K., Dhara S. (2020). First report of substituted 2H-pyranoids from brown seaweed *Turbinaria conoides* with antioxidant and anti-inflammatory activities. Nat. Prod. Res..

[128] Dhara S., Chakraborty K. (2021). Turbinafuranone A–C, new 2-furanone analogures from marine macroalga *Turbinaria ornata* as prospective anti-hyperglycemic agents attenuate tyrosine phosphatase-1B. Med. Chem. Res..

[129] Chakraborty K., Dhara S. (2022). Spirornatas A–C from brown alga *Turbinaria ornata*: Anti-hypertensive spiroketals attenuate angiotensin-I converting enzyme. Phytochemistry.

[130] Qi S.-H., Zhang S., Huang J.-S., Xiao Z.-H., Wu J., Long L.-J. (2004). Glycerol derivatives and sterols from *Sargassum parvivesiculosum*. Chem. Pharm. Bull..

[131] Chang H.W., Jang K.H., Lee D., Kang H.R., Kim T.-Y., Lee B.H., Choi B.W., Kim S., Shin J. (2008). Monoglycerides from the brown alga *Sargassum sagamianum*: Isolation, synthesis, and biological activity. Bioorg. Med. Chem. Lett..

[132] Kim Y.H., Kim E.-H., Lee C., Kim M.-H., Rho J.-R. (2007). Two new monogalactosyl diacylglycerols from brown alga *Sargassum thunbergii*. Lipids.

[133] Cai Y.-P., Xie C.-B., Wang B.-C., Li P.-L., Li B.-F. (2010). Two new resorcinols from *Sargassum thunbergii*. J. Asian Nat. Prod. Res..

[134] Alzarea S.I., Elmaidomy A.H., Saber H., Musa A., Al-Sanea M.M., Mostafa E.M., Hendawy O.M., Youssif K.A., Alanazi A.S., Alharbi M. (2021). Potential anticancer lipoxygenase inhibitors from the Red Sea-derived brown algae *Sargassum cinereum*: An in-silico-supported in-vitro study. Antibiotics.

[135] Peng Y., Cao L., Liu Y., Huang R. (2020). Sargassulfamide A, an unprecedented amide derivative from the seaweed *Sargassum naozhouense*. Chem. Nat. Compd..

[136] Banaigs B., Francisco C., Gonzalez E., Codomier L. (1984). Lipids from the brown alga *Cystoseira barbata*. Phytochemistry.

